# A Wireless 3D Magneto‐Mechanical Stimulation Platform Drives In Situ Chondrogenic Commitment of Endogenous MSCs

**DOI:** 10.1002/advs.75958

**Published:** 2026-06-04

**Authors:** Zhenguang Li, Li Peng, Laiya Lu, Yingying Wang, Xiaolei Chen, Feng Yin, Cuijun Deng, Yu Cheng

**Affiliations:** ^1^ Shanghai Key Laboratory of Anesthesiology and Brain Functional Modulation Clinical Research Center For Anesthesiology and Perioperative Medicine Translational Research Institute of Brain and Brain‐Like Intelligence Shanghai Fourth People's Hospital School of Medicine Tongji University Shanghai China; ^2^ State Key Laboratory of Autonomous Intelligent Unmanned Systems Tongji University Shanghai China; ^3^ Department of Joint Surgery Shanghai East Hospital, School of Medicine Tongji University Shanghai China

**Keywords:** chondrogenesis, magnetic fields, magnetic nanomotors, mechanical signals, mesenchymal stem cells

## Abstract

Mechanical signals play a fundamental role in regulating stem cell fate. However, due to the lack of spatiotemporal mechanical tools, directing the in situ chondrogenic differentiation of endogenous bone marrow mesenchymal stem cells (BMSCs) remains a formidable challenge in articular cartilage repair. Here, we develop a magneto‐mechanical stimulation platform with unique 3D actuation to orchestrate chondrogenic commitment of endogenous BMSCs. The BMSC‐targeted antioxidative magnetic nanomotors are designed to perform trans‐planar rotational‐bouncing motions in lysosomes under a self‐developed 3D rotating fluctuating magnetic field, generating amplified dynamic mechanical stimulation. They potentiate chondrogenic differentiation of BMSCs by lysosomal mechanically‐gated actin cytoskeletal remodeling. After intra‐articular delivery of nanomotors via an injectable hydrogel, they can target endogenous BMSCs and be wirelessly controlled to apply the dynamic mechanical stimulation in vivo for hyaline cartilage regeneration. Beyond cartilage repair, this platform offers a generalized methodology to remotely regulate the mechanical signals of cell fate for regenerative medicine.

## Graphical abstract

An intracellular magneto‐mechanical platform utilizing MSC‐targeted nanomotors is developed. After intra‐articular delivery, these nanomotors target endogenous BMSCs and are actuated within lysosomes to execute trans‐planar rotational‐bouncing motions under a rotating‐fluctuating 3D magnetic field, generating amplified mechanical stimulation. This platform acts as a programmable mechanical switch, reconfiguring the actin cytoskeleton to orchestrate the BMSC chondrogenic fate for enhanced in situ cartilage regeneration.

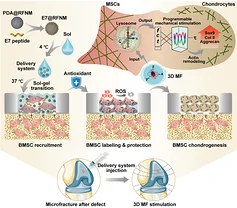

## 1 Introduction

Mesenchymal stem cells (MSCs) possess the intrinsic potential for self‐renewal and multilineage differentiation [1], making them pivotal candidates for regenerating load‐bearing tissues like articular cartilage [2, 3]. Harnessing the regenerative potential of endogenous MSCs represents a promising alternative to the complex and costly transplantation of exogenous cells [4, 5]. While exogenous cell therapies have been advanced, they are often hampered by low survival rates, immune rejection risks, and intricate regulatory hurdles [6]. In contrast, endogenous MSCs are naturally adapted to the host environment [7]. However, successfully leveraging these native cells for articular cartilage repair remains elusive. Although techniques like the microfracture can mobilize endogenous BMSCs to the defect sites, they typically yield the fibrocartilaginous tissue instead of hyaline cartilage due to the lack of precise control over their differentiation in the hostile inflammatory microenvironments [8, 9].

To achieve cartilage repair, active regulation for in situ chondrogenic commitment is required, besides sufficient mobilization of endogenous MSCs. While biochemical cues have been extensively studied, mechanical cues as fundamental physical regulators of cell fate offer a more localized control modality [10, 11]. Particularly, articular cartilage, as a highly specialized load‐bearing tissue, relies inherently on dynamic mechanical cues to maintain its structural and functional homeostasis [12, 13]. Consequently, mimicking physiologically relevant mechanical environments has been a dominant strategy to guide the chondrogenic commitment of MSCs [14, 15, 16]. For instance, various types of bio‐scaffolds and mechanical loading systems have shown positive effects on MSCs in terms of chondrogenic gene expression and matrix protein synthesis by tailoring mechanical properties (viscoelasticity, relaxation, topography, etc.) or applying mechanical stress (compression, tension, shear stress, and hydrostatic pressure) [17, 18, 19]. However, these extracellular strategies face significant translational hurdles in the modulation of endogenous MSCs due to a lack of flexibility and spatiotemporal precision required for controlled mechanical intervention at the subcellular level. Developing a wireless, high‐fidelity technique to remotely and precisely actuate intracellular mechanics could represent a paradigm shift in programming stem cell fate in situ.

Magneto‐mechanical actuation has emerged as a powerful tool for such remote modulation, leveraging the dynamic motion of magnetic nanomotors as wireless transducers to convert external magnetic fields (MF) into localized mechanical forces or torques [20, 21]. With their nanoscale dimensions and functionalized surfaces, magnetic nanomotors can selectively target subcellular structures to achieve precise regulation of specific signaling pathways under the actuation of the external MF [22, 23], providing deep tissue penetration and exceptional spatiotemporal controllability [24, 25]. Conventionally, magneto‐actuated propulsion typically relies on the modes of planar translational or rotational motion under 2D gradient or rotating MF [26, 27, 28]. However, in the highly viscous, non‐Newtonian environment of the intracellular cytoplasm, these 2D motions with low degrees of freedom struggle to generate sufficient dynamic mechanical stimulation. Increasing the nanomotor dosage or elevating the MF parameters are thus forced to achieve effective mechanotransduction, which raises widespread biosafety concerns or a high requirement of the MF device. We posit that the integration of multiple motion modalities of magneto‐actuated nanomotors with high degrees of freedom could fulfill the mission of intracellular mechanotransduction with high efficiency.

**FIGURE 1:**
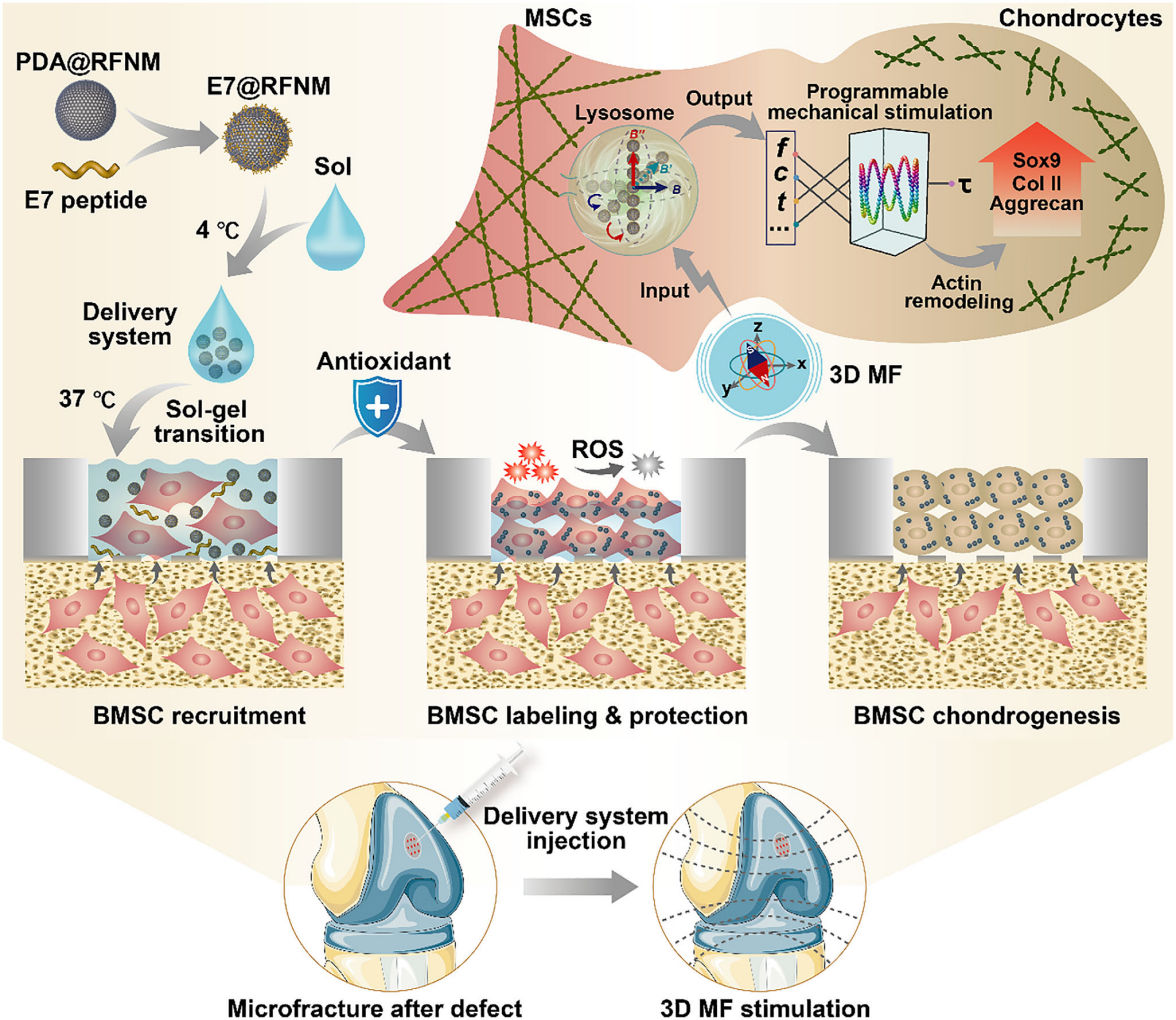
Schematic of the antioxidative nanomotor‐mediated intracellular 3D magneto‐mechanical stimulation platform for in situ chondrogenic programming of endogenous BMSCs and enhancing cartilage repair. The therapeutic effect of cartilage repair was achieved through the following three stages: (i) the intra‐articular sustained release of nanomotors from the delivery system enabled them to recruit and target the microfracture‐liberated endogenous BMSCs, (ii) the antioxidative ability of nanomotors conferred endogenous BMSCs the defense against oxidative stress, (iii) the programmable magneto‐mechanical stimulation from intracellular multimodal motion of nanomotors under the remote actuation of the 3D MF propelled the chondrogenic differentiation of BMSCs through inducing actin cytoskeleton remodeling.

Herein, we proposed a wireless magneto‐mechanical stimulation platform that leveraged the multimodal motion of nanomotors to orchestrate the in situ chondrogenic commitment of endogenous BMSCs. Antioxidative magnetic nanomotors (PDA@RFNMs) that were modified with the MSC‐specific affinity peptide (E7 peptide) [29] were designed and delivered intra‐articularly, taking thermosensitive hydrogels as carriers. These delivered nanomotors enabled sustained release to recruit and target the microfracture‐liberated endogenous BMSCs while protecting them from oxidative stress. Crucially, by self‐developing a rotating fluctuating 3D MF, we overcame the degree‐of‐freedom limitations of magnetic actuation and elevated it to five degrees of freedom. This system remotely actuated lysosome‐internalized nanomotors to execute a trans‐planar rotational‐bouncing motion for significantly amplified dynamic mechanical output by programmable MF control. We demonstrated that this magneto‐mechanical perturbation acts as a potent physical switch for programming chondrogenic fate of BMSCs through a mechanism involving lysosomal mechanically‐gated dynamic actin cytoskeleton remodeling. As a proof‐of‐concept for this “mechanical programming” strategy, the platform is validated in a rat in situ cartilage regeneration model (Figure 1).

## 2 Results

### 2.1 Design and Characterizations of the Antioxidative Magnetic Nanomotors

**FIGURE 2:**
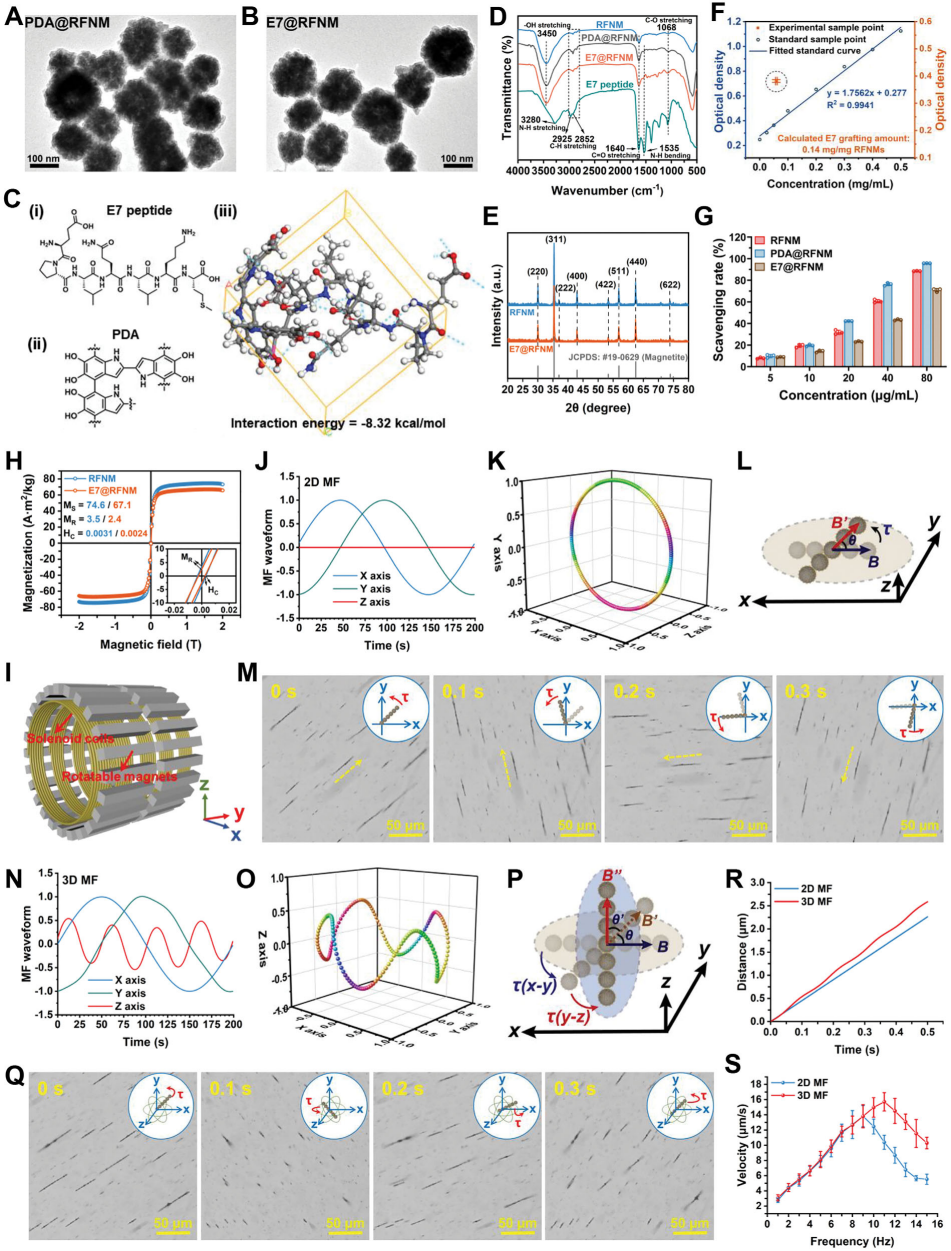
Characterizations and magneto‐actuated motion control of nanomotors. (A, B) Representative TEM images of PDA@RFNMs and E7@RFNMs. (C) The interaction energy between the E7 peptide and PDA was calculated by the molecular docking simulation. (D) FTIR spectra of nanomotors. (E) XRD patterns of nanomotors. (F) E7 grafting amount on RFNMs calculated using the standard curve. (G) DPPH· scavenging rates of nanomotors. (H) Magnetic hysteresis curves of nanomotors and their zoomed‐in view of the central region (inset). The values of saturation magnetization (M_S_), remanent magnetization (M_R_), and coercive field (H_C_) were marked there. (I) Schematic diagram of the construction of the magnetic actuation system. (J, K) Configuration of the 2D MF generated by the periodic electromagnetic signals along the x‐y axes. (L) Schematic illustration of the planar motion of nanomotors in response to the 2D MF. (M) Motion control of nanomotors under the actuation of the 2D MF (20 mT, 1 Hz). (N, O) Configuration of the 3D MF generated by the x‐y‐z triaxial periodic electromagnetic signals. (P) Schematic illustration of trans‐planar motion of nanomotors in response to the 3D MF. (Q) Motion control of nanomotors under the actuation of the 3D MF (20 mT, [1, 1] Hz). (R) COMSOL Multiphysics‐simulated travel distance of nanomotors within one cycle in response to the 2D MF and 3D MF. (S) Travel velocity of nanomotors under the actuation of different frequencies of the 2D MF and 3D MF.

The design of the antioxidative magnetic nanomotors was approached from two key aspects. The Rhein@Fe_3_O_4_ nanomotors (RFNMs) with the magneto‐responsive and antioxidative properties were firstly synthesized through a typical solvothermal procedure taking an anthraquinone compound rhein as the ligand [30]. The RFNMs were then coated with polydopamine (PDA) to serve as the anchoring sites for non‐covalent adsorption of the E7 peptide, allowing them to specifically recruit and target endogenous BMSCs (Figure S1A) [31]. The stepwise surface modification of nanomotors was displayed from a bare spherical cluster (Figure S1B) to a thin film‐coated structure (Figure 2A,B) with a final diameter of ∼180.7 nm (Figure S1C), and also evidenced by incremental increases in hydrodynamic diameter (from 223.1 nm of RFNMs to 266.4 nm of PDA@RFNMs to 281.4 nm of E7@RFNMs) (Figure S1D) as well as shifts in zeta potential (from −4.04 mV of RFNMs to −15.6 mV of PDA@RFNMs to −9.66 mV of E7@RFNMs) (Figure S1E). The calculated interaction energy of −8.32 kcal/mol indicated a strong non‐covalent binding that primarily mediated by hydrogen bonding and electrostatic interactions between PDA and the E7 peptide (Figure 2C). The chemical structure and composition were further validated by multiple characterizations. Fourier‐transform infrared (FTIR) spectrum of E7@RFNMs revealed characteristic peaks of catechol, amino, and amide groups from PDA and E7 peptide (Figure 2D). X‐ray diffraction (XRD) patterns corresponded to the typical face‐centered cubic structure of magnetite (Figure 2E). X‐ray photoelectron spectroscopy (XPS) confirmed the elemental composition (C, N, O, Fe) and identified the valence states of iron (Fe^2+^/Fe^3+^) and various forms of bound oxygen (Fe─O, C─O, C═O) (Figure S1F–H). The loading capacity of E7 peptide on the surface of nanomotors were quantified as 0.14 mg/mg RFNMs (Figure 2F).

Furthermore, the antioxidative ability of the nanomotors was verified by evaluating the radical scavenging activity. Both rhein and PDA as the common polyphenolic antioxidants, are known to block the radicals that resulted from the ROS‐initiated autoxidation of cellular biomacromolecules via the hydrogen atom transfer mechanism (Figure S2A) [32]. All nanomotors exhibited concentration‐dependent radical scavenging effects, evidenced by the progressive color fading of colors of the DPPH· solution (Figure S2B) and a corresponding decrease in its characteristic absorbance in UV–vis spectra (Figure S2C–E). Notably, PDA@RFNMs showed an enhanced radical scavenging activity over RFNMs due to a synergistic contribution of PDA and rhein. However, subsequent modification of the E7 peptide significantly attenuated this activity, likely by shielding partially the active polyphenolic sites (Figure 2G).

### 2.2 Motion Control of the Nanomotors in Response of the 3D MF

The RFNMs presented a high saturation magnetization (M_S_) value of 74.6 A·m^2^/kg, and the saturation magnetization value of E7@RFNMs was shifted to 67.1 A·m^2^/kg due to the surface modification. Before and after the surface modification, the nanomotors exhibited extremely low remanent magnetization (M_R_) and coercive field (H_C_), indicating their superparamagnetic properties (Figure 2H). High‐efficiency conversion of magnetic energy into the mechanical output requires intelligent coupling of magnetic nanomotors and a magnetic actuation system. A self‐designed magnetic actuation system was developed to control the multimodal motion of nanomotors. This system coupled an axial electromagnetic field (EMF, x Hz) with oscillatory mode and a radial permanent magnetic field (PMF, y Hz) with rotational mode to generated the rotating fluctuating 3D MF ([x,y] Hz), enabling multi‐degree‐of‐freedom motion control of magnetic nanomotors within a large workspace with 30 cm diameter (Figure 2I and Figure S3A). When exposed to the 3D MF (20 mT, [1, 1] Hz), the nanomotors underwent real‐time magneto‐guided self‐assembly and gradually aligned into chains due to the magnetic dipole‐dipole interactions (Figure S3B). The chain length increased as the duration of 3D MF prolonged (Figure S3C).

Importantly, the nanomotors exhibited programmable responsiveness to different MF configurations, allowing precise control of motion modes and mechanical outputs. Under a rotating two‐dimensional MF (2D MF, 20 mT, 1 Hz) created by the periodic electromagnetic signals along the x‐y axes (Figure 2J,K), the nanomotors presented the planar rotational motion to output two‐dimensional magnetic torques confined within the x‐y plane (Figure 2L‚M and Movie S1). In contrast, exposure to the 3D MF (20 mT, [1, 1] Hz) tuned by synchronized x‐y‐z triaxial periodic electromagnetic signals (Figure 2, N,O) allowed the nanomotors being actuated in five degrees of freedom to exhibit trans‐planar rotational‐bouncing motion to output magnetic torques (Figure 2P,Q and Movie S2).

The nanomotor motions in response to the 2D and 3D MF were analyzed by a theoretical dynamic model. It demonstrated that the 3D MF‐actuated nanomotors achieved a greater travel displacement with a 15.5% increase of velocity than 2D MF‐actuated nanomotors at the same frequency (Figure 2R). Moreover, the propulsion velocity of nanomotors gradually increased with the applied MF frequency until reaching the out‐of‐step threshold. The out‐of‐step frequency of the 3D MF‐actuated nanomotors (11 Hz) was 22.2% higher than that of 2D MF‐actuated nanomotors (9 Hz, Figure 2S). These results indicated that the 3D MF with high degree‐of‐freedom actuation provided an enhanced propulsion efficiency for the magnetic nanomotors.

### 2.3 Intracellular Magneto‐Mechanical Actuation of Nanomotors in Response of the 3D MF

**FIGURE 3:**
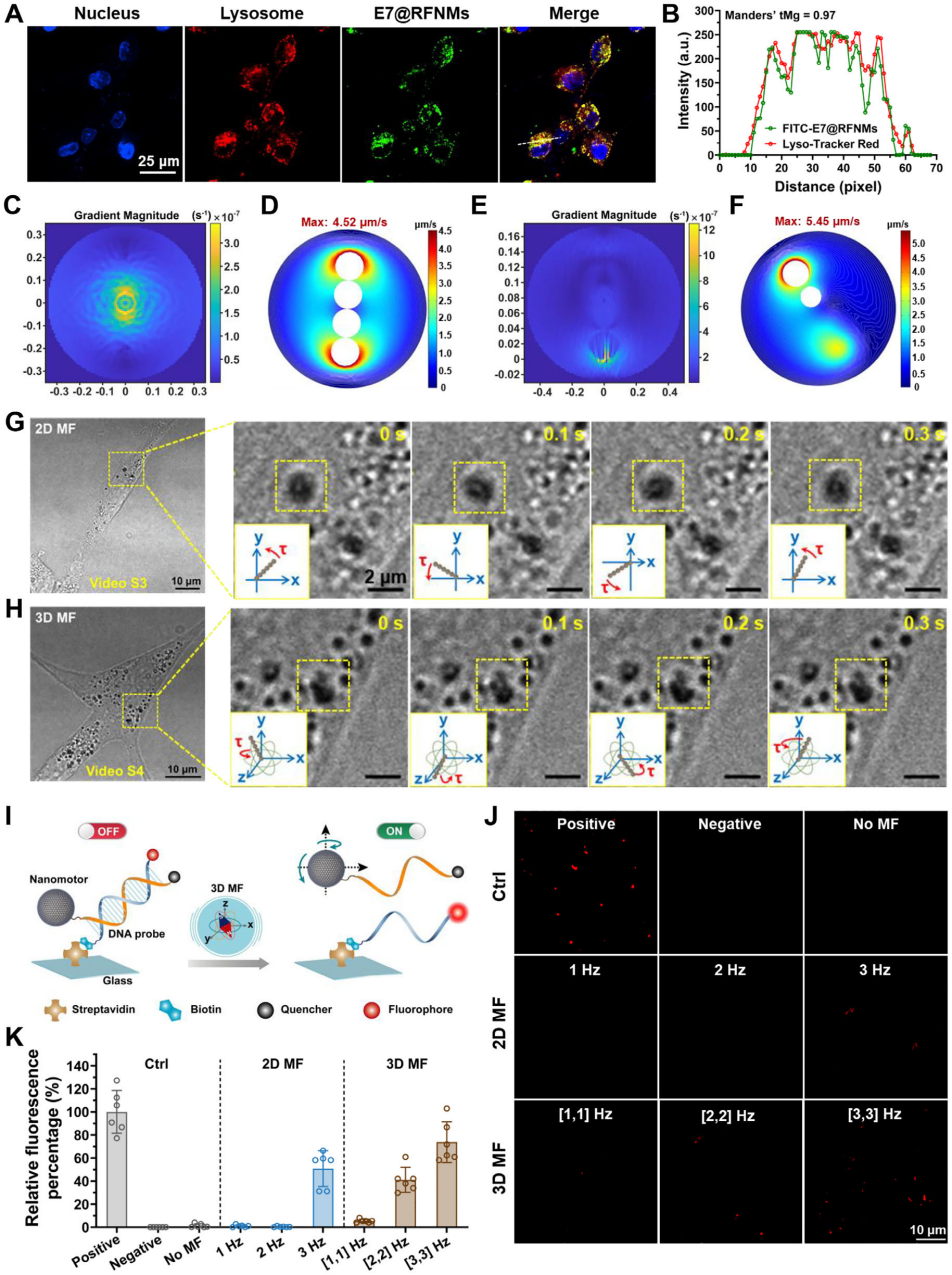
Intracellular magneto‐mechanical actuation of nanomotors. (A) Confocal micrographs of BMSCs after coculturing with 10 µg/mL of FTIC‐E7@RFNMs and stained with Lyso‐Tracker Red and Hoechst. (B) Representative fluorescence profiles of FITC‐E7@RFNMs and Lyso‐Tracker Red from the white line in merge image. (C, D) COMSOL Multiphysics‐simulated flow field gradient magnitude and fluid velocity induced by nanomotor motion in response to the 2D MF. (E, F) COMSOL Multiphysics‐simulated flow field gradient magnitude and fluid velocity induced by nanomotor motion in response to the 3D MF. (G, H) Visualized motion of nanomotors inside lysosomes of BMSCs under the actuation of the 2D MF and 3D MF. (I) Schematic illustration of a force‐sensitive DNA probe detecting magneto‐mechanical output from 3D MF‐actuated nanomotors. (J) Representative fluorescence images of the nanomotor‐DNA probe system after exposure to different frequencies of 2D MF and 3D MF. (K) Quantitative relative fluorescence percentage using the fluorescence images of the nanomotor‐DNA probe system (n = 6).

Following coculture with BMSCs, the confocal imaging revealed that most of nanomotors were localized in lysosomes with a Manders’ coefficient of 0.97, suggesting the strong colocalization (Figure 3A,B). It is assumed that the magnetic torques generated by the intracellular nanomotor assembly and motion in response to the external MF could induce vortex flow of intracellular fluid. Finite element simulation results of the lysosomal fluid velocity using COMSOL Multiphysics showed that the flow gradient (∼12 × 10^−7^ s^−1^) and maximum flow velocity (5.45 µm/s) caused by the magnetic torques output from the 3D MF actuated‐nanomotors (Figure 3C,D), was significantly higher than those under the 2D MF (∼3 × 10^−7^ s^−1^ and 4.52 µm/s) (Figure 3E,F). Higher flow gradient of the intracellular fluid implied larger shear stress to the intracellular components.

The real‐time motion of the 2D MF and 3D MF actuated‐nanomotors inside the BMSCs was further visualized using confocal microscopy. Consistent with the results in acellular conditions (Figure 2M,Q), the nanomotors inside cellular lysosomes exhibited distinct motion patterns depending on the MF configurations. As depicted in the time‐lapse sequence, the nanomotors executed a constrained rotational motion on a fixed axis within the focal plane under the 2D MF (Figure 3G and Movie S3). In contrast, the application of 3D MF induced a trans‐planar rotational‐bouncing motion of nanomotors, dynamically reorienting itself in 3D space within the lysosomes. This sophisticated motion implied the generation of magnetic torques capable of applying mechanical stimulation in multiple directions (Figure 3H and Movie S4). These results confirmed that the nanomotors acted as precise intracellular actuators, capable of delivering tunable dynamic mechanical cues directly to subcellular structures to modulate cell functions.

Besides, a force‐sensitive DNA probe with a melting threshold of 12 pN was employed to display the magneto‐mechanical output of the MF‐actuated nanomotors. The fluorescence‐labeled DNA strands were immobilized on glass substrates through biotin‐streptavidin coupling, and their complementary quencher strands were conjugated to the nanomotor surface. Upon switching the 3D MF from “OFF” to “ON”, the nanomotors could mechanically melt the DNA probe, thereby restoring fluorescence (Figure 3I). Negligible fluorescence signals were detected under the 2D MF actuation at 2 Hz, while obvious fluorescence restoration (∼41.1%) occurred at [2, 2] Hz after the same time of 3D MF actuation. When the 2D MF increased to 3 Hz, a modest fluorescence activation (∼50.7%) was observed, but further intensified (∼73.8%) under the 3D MF actuation at [3, 3] Hz (Figure 3J,K), demonstrating a superior efficiency of magnetic‐mechanical conversion at equivalent input frequencies. These results confirmed that the 3D MF actuation enabled nanomotors to output enhanced mechanical torque at lower external inputs, indicating their potential as an efficient nanoscale platform for intracellular mechanical stimulation.

### 2.4 Magneto‐Mechanical Programming of Chondrogenic Differentiation of BMSCs

Dynamic mechanical stimulation has been well‐established in modulating the chondrogenesis of stem cells [13, 18, 33]. We assumed that the magneto‐mechanical stimulation generated from the coupling of intracellular nanomotors and the 3D MF could program the chondrogenic fate of BMSCs, necessitating the clarification of the magnetic torque‐dependent effect. The compatibility of nanomotors with BMSCs was investigated to optimize the nanomotor concentration for magneto‐mechanical modulation. After 24 h of coculture with varying concentrations of nanomotors (E7@RFNMs and PDA@RFNMs), the BMSCs were able to effectively phagocytize the nanomotors in a dose‐dependent manner, with a notably higher uptake of E7@RFNMs than that of PDA@RFNMs at the same concentration, reflecting the high affinity of the grafted E7 peptide to the BMSCs (Figure S4A,B). However, the loading efficiency of E7@RFNMs decreased with increasing feed mass, probably due to the saturation of cellular uptake in contrast to the continuously increased loading efficiency of PDA@RFNMs (Figure S4C). As a result, the E7@RFNMs showed a higher toxicity to BMSCs than the PDA@RFNMs, especially at concentrations exceeding 20 µg/mL (Figure S5A). Various concentrations of E7@RFNMs did not inhibit the proliferation of BMSCs in a period of 7 days of coculture (Figure S5B,C). Moreover, the impact of magnetic‐mechanical stimulation on BMSC growth was evaluated under different frequencies of the 3D MF (10 min/day). As the concentration of E7@RFNMs and the 3D MF frequency increased, the inhibitory effect of the magnetic‐mechanical stimulation on cell growth also increased because of the enhancement of the magneto‐mechanical output (Figure S6A–C). These results suggested that the concentrations below 10 µg/mL of E7@RFNMs were suitable for subsequent experimental studies.

**FIGURE 4:**
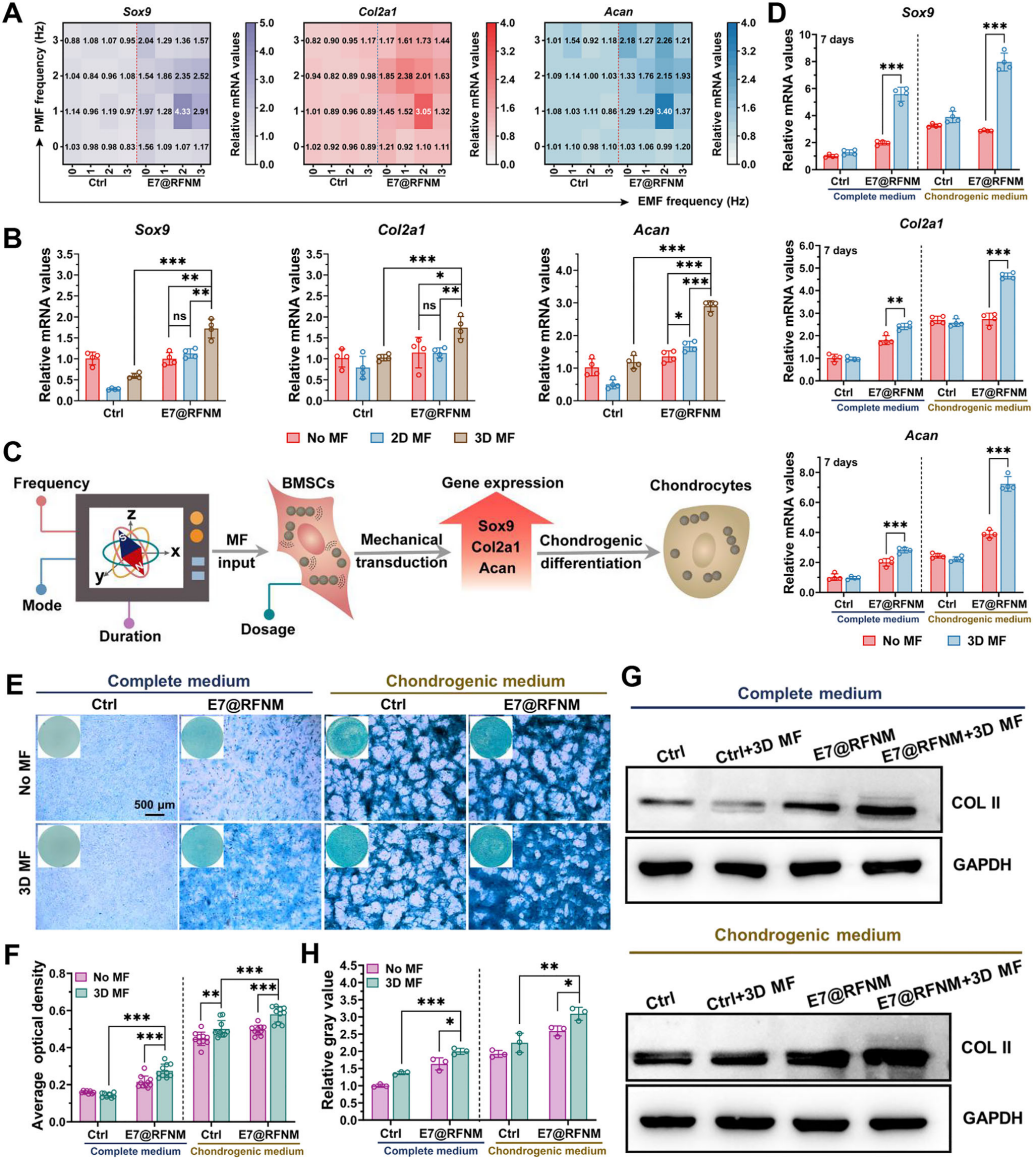
Magneto‐mechanical programming of chondrogenic differentiation of BMSCs. (A) The expression of chondrogenic genes of BMSCs labeled with 10 µg/mL of E7@RFNMs under the actuation of different frequencies of 3D MF (75 mT, 10 min/day) for 3 days (n = 4). (B) The expression of chondrogenic genes of BMSCs labeled with 10 µg/mL of E7@RFNMs under the actuation of the 2D MF (75 mT, 2 Hz, 10 min/day) and the 3D MF (75 mT, [2, 1] Hz, 10 min/day) for 3 days (n = 4, ^*^p < 0.05, ^*^ ^*^p < 0.01 and ^**^ ^*^p < 0.001). (C) Schematic illustration of programmable 3D MF parameters outputting dynamic magneto‐mechanical stimulation that propels the chondrogenic differentiation of BMSCs. (D) The expression of chondrogenic genes of BMSCs labeled with 10 µg/mL of E7@RFNMs in complete and chondrogenic medium under the 3D MF actuation for 7 days (n = 4, ^*^ ^*^p < 0.01 and ^**^ ^*^p < 0.001). (E) Alcian blue staining of BMSCs labeled with 10 µg/mL of E7@RFNMs in complete and chondrogenic medium under the 3D MF actuation for 7 days. (F) Quantitative average optical density from the Alcian blue staining images (n = 10, ^*^ ^*^p < 0.01 and ^**^ ^*^p < 0.001). (G) COL II protein expression of BMSCs labeled with 10 µg/mL of E7@RFNMs in complete and chondrogenic medium under the 3D MF actuation for 7 days. (H) Quantitative relative gray values from the Western‐blot analysis (n = 3, ^*^p < 0.05, ^*^ ^*^p < 0.01, and ^**^ ^*^p < 0.001).

Next, the programming effect of intracellular magneto‐mechanical modulation on the chondrogenic commitment of BMSCs was systematically investigated with the input variables of nanomotor dosage, 3D MF frequency, 3D MF duration time, as well as MF mode by detecting the expression of specific chondrogenic genes (*Sox9*, *Col2a1*, and *Acan*). Notably, the gene expression in 10 µg/mL of E7@RFNM‐labeled BMSCs was overall higher than that in the control group and 5 µg/mL group under the actuation of different 3D MF frequencies with a fixed duration time of 10 min (Figure S7A). These results suggested that the opportune dynamic magneto‐mechanical stimulation served as a positive regulator of chondrogenic differentiation of BMSCs. Subsequently, the 3D MF frequency as a combination of EMF frequency and PMF frequency was optimized with a fixed E7@RFNM concentration of 10 µg/mL and a fixed duration time of 10 min. The gene expression in E7@RFNM‐labeled BMSCs showed significant upregulation at specific 3D MF frequencies when compared with the control groups, indicating that a specific resonance frequency may exist to activate specific gene expression. Notably, a 3D MF frequency of [2, 1] Hz overall induced the highest upregulated expression of chondrogenic genes in BMSCs (Figure 4A). Then, under a fixed E7@RFNM concentration of 10 µg/mL and a fixed 3D MF frequency of [2, 1] Hz, the gene expression was gradually increased as the daily 3D MF duration time until it leveled off when the 3D MF was applied over 10 min/day (Figure S7B).

Furthermore, the 3D MF outperformed the 2D MF in propelling chondrogenic differentiation of BMSCs under the equivalent input parameters, ascribing to the enhanced magnetic‐mechanical conversion from intracellular multi‐dimensional motion of nanomotors (Figure 4B). It also means that the 3D MF with the multi‐degree‐of‐freedom actuation could achieve efficient mechanical manipulation of cellular functions with minimal external inputs, which could reduce the risk of adverse effects and hardware cost of the MF setup. Therefore, the 3D MF‐actuated multimodal motion of nanomotors represented a programmable mechanical intervention strategy by which the BMSCs could be modulated with personalized parameter combinations (dosage, frequency, mode, and duration) to activate mechanosensitive pathways for chondrogenic differentiation (Figure 4C). From the above results, a set of 3D MF parameters (10 µg/mL, [2, 1] Hz, and 10 min/day) was identified as the optimal input index for magneto‐mechanically intervening in chondrogenic commitment of BMSCs. With these input parameters, the facilitating effect of magneto‐mechanical stimulation on the chondrogenic differentiation of BMSCs was eventually determined with significantly upregulated expression of chondrogenic genes after a culture period up to 7 days in complete medium, and especially in chondrogenic medium (Figure 4D and Figure S8).

The promoting effect of intracellular nanomotor‐mediated magneto‐mechanical stimulation on the chondrogenic commitment of BMSCs was further validated by investigating the synthesis of the cartilage‐specific matrix components glycosaminoglycan (GAG) and type II collagen (COL II). Consistent with the results of gene expression, the Alcian blue staining and quantitative analysis showed that significantly enhanced blue signals were observed in the 3D MF‐actuated BMSCs compared to other groups no matter in complete or chondrogenic medium, indicating that 3D MF‐generated dynamic mechanical stimulation was able to accelerate the synthesis of GAG (Figure 4E,F). Similarly, the 3D MF‐actuated BMSCs showed stronger fluorescent signals of COL II than other groups in both complete and chondrogenic media from the immunofluorescence staining and quantitative analysis (Figure S9A,B). Convincingly, the Western blot analysis and quantitative results also proved that the secretion of COL II in the 3D MF‐actuated BMSCs was obviously higher than that in the control groups in both complete and chondrogenic media (Figure 4G,H). It is also noteworthy that the cartilage‐specific matrix protein synthesis of 3D MF‐actuated BMSCs in chondrogenic medium was remarkably more than that in complete medium because of the cooperative regulation of specific biochemical factors and dynamic mechanical stimulation. Collectively, these results provided ample evidence that the dynamic magneto‐mechanical stimulation from 3D MF‐actuated intracellular motion of nanomotors could serve as an intelligent and robust manipulation tool for promoting the chondrogenic differentiation of BMSCs.

### 2.5 Underlying Mechanism of Magneto‐Mechanical Modulated Chondrogenic Differentiation of BMSCs

**FIGURE 5:**
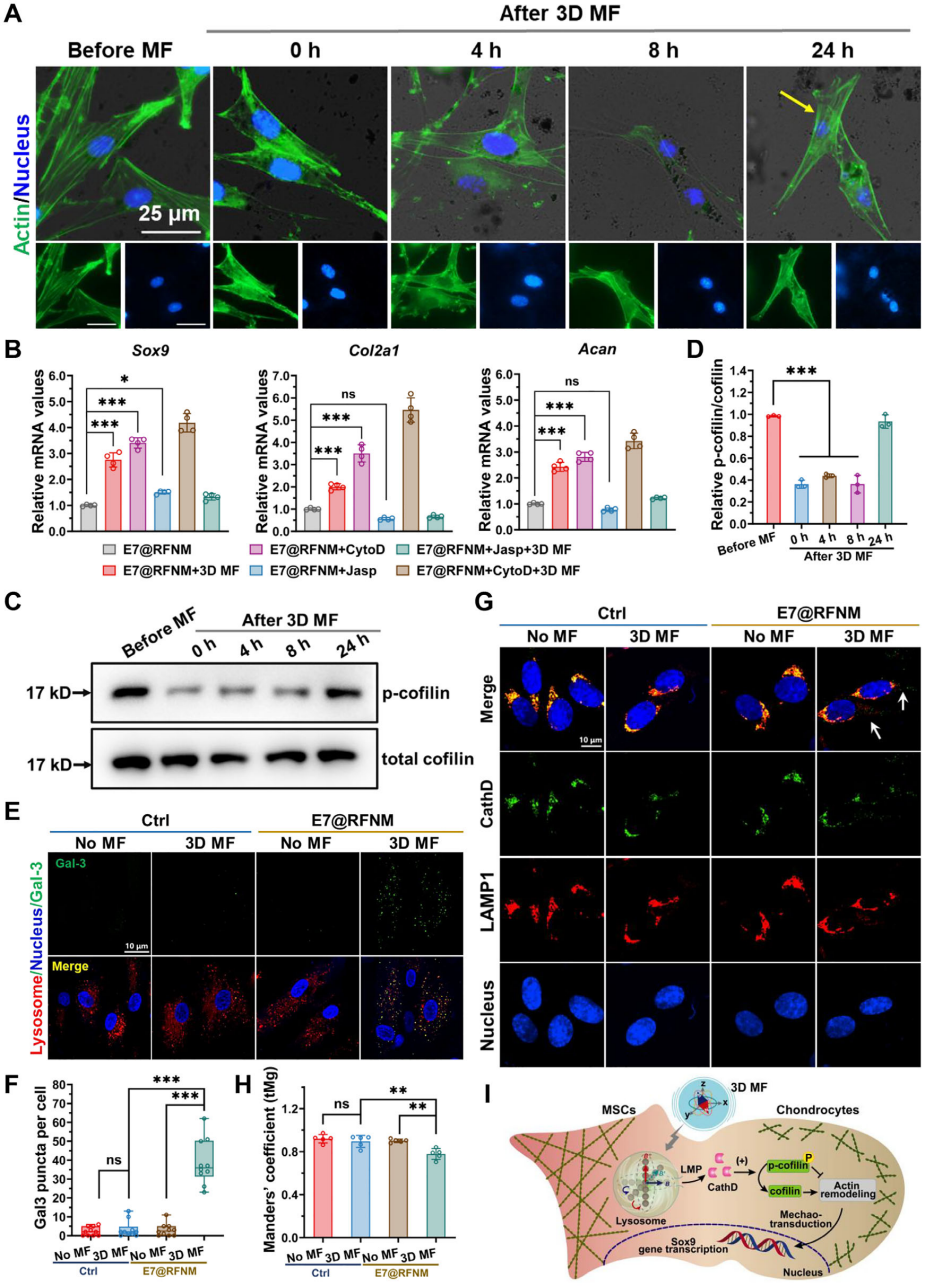
Underlying mechanism of magneto‐mechanical modulated chondrogenic differentiation of BMSCs. (A) Actin staining of BMSCs labeled with 10 µg/mL of E7@RFNMs at different time points before and after the 3D MF actuation. (B) The expression of chondrogenic genes of BMSCs labeled with 10 µg/mL of E7@RFNMs in the presence and absence of CytoD or Jasp under the 3D MF actuation for 3 days (n = 4, ^*^p < 0.05, and ^**^ ^*^p < 0.001). (C) Cofilin and p‐cofilin protein expression of BMSCs labeled with 10 µg/mL of E7@RFNMs at different time points before and after the 3D MF actuation. (D) Quantitative relative p‐cofilin level from the Western‐blot analysis (n = 3, ^**^ ^*^p < 0.001). (E) Fluorescence images of BMSCs transfected with the EGFP‐Gal3 plasmid and labeled with 10 µg/mL of E7@RFNMs after the 3D MF actuation. (F) Statistical counts of Gal3 puncta per cell (n = 10, ^**^ ^*^p < 0.001). (G) CathD and LAMP1 immunofluorescence staining of BMSCs labeled with 10 µg/mL of E7@RFNMs after the 3D MF actuation. (H) Manders’ coefficient (tMg) for colocalization of CathD with lysosomes. (I) Schematic illustration of lysosomal mechanically gated actin remodeling under the 3D MF actuation to propel chondrogenic differentiation of BMSCs.

The chondrogenic fate of MSCs involves dynamic changes in cell morphology, which is precisely regulated by cytoskeletal rearrangements [34, 35, 36]. Previous studies have reported that biochemical stimuli of cytochalasin D (CytoD, an actin polymerization inhibitor) [37] and biophysical stimuli of continuous low‐intensity ultrasound [38] could upregulate Sox9 expression through disruption of actin organization, thereby promoting chondrogenic lineage commitment of MSCs. In this study, the results of actin staining showed that the 3D MF mechanical stimulation caused the depolymerization of actin microfilaments. But the disrupted actin filaments were observed to be repolymerized at 24 h after the 3D MF mechanical stimulation, implying the necessity of daily stimulation for sustained chondrogenic commitment of MSCs (Figure 5A). Additionally, the CytoD further enhanced the 3D MF mechanical stimulation‐induced expression upregulation of chondrogenic genes, whereas the Jasplakinolide (Jasp, an actin polymerization inducer) inhibited the promoting effect of 3D MF mechanical stimulation on the gene expression, validating that actin cytoskeletal remodeling induced by dynamic magneto‐mechanical stimulation is the primary event in propelling BMSC differentiation (Figure 5B). Then, we investigated the phosphorylation level of cofilin, an actin‐depolymerizing factor whose activity is inhibited by the phosphorylation of the Ser3 (N‐terminal serine) site [39]. Our results showed an immediate and sustained downregulation in cofilin phosphorylation with significantly decreased p‐cofilin/total cofilin ratio from 0 to 8 h post 3D MF stimulation, which recovered after 24 h (Figure 5C,D). The results coincide with the observed actin reorganization, indicating that the dynamic magneto‐mechanical stimulation‐induced actin remodeling is mediated by dephosphorylation of cofilin.

Previous studies have reported that the cytosolic translocation of cathepsin D (CathD) due to lysosomal membrane permeabilization (LMP) enables it to execute a noncanonical cofilin phosphatase function in a neutral pH environment, mediating actin remodeling by dephosphorylation and activation of cofilin [40, 41]. We engineered enhanced green fluorescent protein‐tagged galectin‐3 (Gal3) plasmids to detect the lysosome permeability, taking Gal3 puncta as indicators. Our results demonstrated that 3D MF mechanical stimulation (E7@RFNM + 3D MF group) triggered LMP with significantly increased Gal3 puncta when compared to control groups (Figure 5E,F). Meanwhile, accelerated movement of lysosomes concomitant with the LMP was also observed under the 3D MF‐mediated mechanical perturbation (Figure S10), which is consistent with the phenomena reported in the literature [42]. Next, immunofluorescence staining was used to investigate the LMP‐induced cytosolic translocation of CathD under the 3D MF stimulation. Without mechanical stimulation (Ctrl, Ctrl + 3D MF, and E7@RFNM groups), CathD colocalized strongly with lysosome‐associated membrane protein type 1 (LAMP1)‐labeled lysosomes. After 3D MF mechanical stimulation (E7@RFNM + 3D MF group), the fluorescence puncta of CathD were observed in the distal area and non‐colocalized with lysosomes, indicative of CathD escape from lysosomes during dynamic magneto‐mechanical stimulation‐triggered LMP (Figure 5G,H). Finally, we proposed the underlying mechanism of magneto‐mechanical modulated chondrogenic differentiation of BMSCs based on the lysosomal gating. 3D MF actuated nanomotors execute multimodal motion in lysosomes, generating dynamic mechanical disturbance for triggering LMP. The lysosomal mechanoporation induces the release of CathD from lysosomes into the cytoplasm, directly dephosphorylating cofilin. The cofilin‐mediated actin remodeling propels the chondrogenic gene transcription by activating cytoskeletal‐nuclear mechanotransduction pathways (Figure 5I).

### 2.6 In Situ Magneto‐Mechanical Modulation of Endogenous BMSCs for Enhanced Cartilage Repair

**FIGURE 6:**
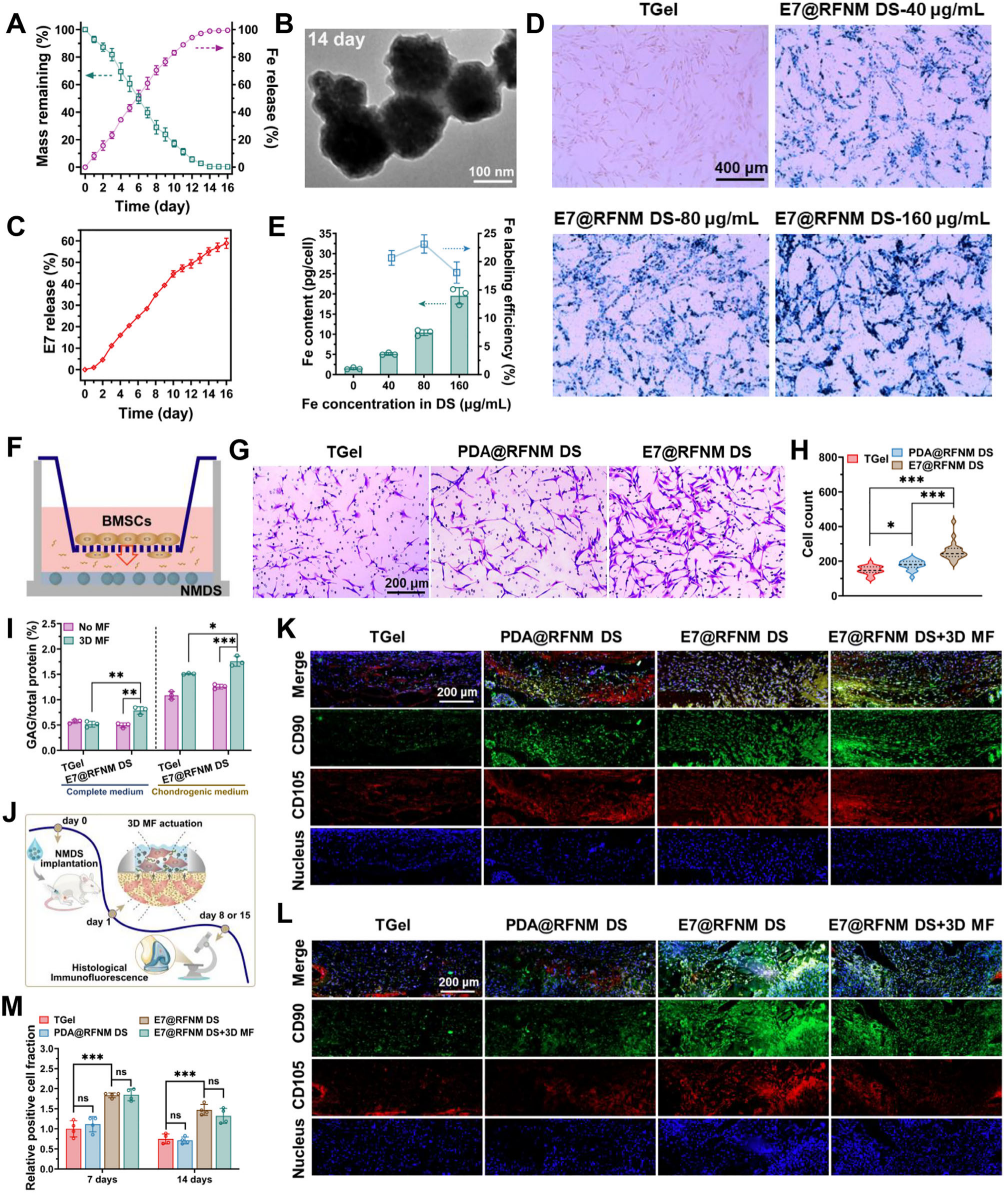
BMSC recruitment, labeling, and chondrogenic modulation of the NMDS. (A) The dissociation curve of the NMDS and the release profile of nanomotors from the NMDS in vitro. (B) TEM image of the nanomotors released at day 14. (C) The release profiles of the E7 peptide from the NMDS in vitro. (D) PB & NR staining of BMSCs after coculturing with different concentrations of E7@RFNMs loaded NMDS for 24 h. (E) Quantitative uptake of nanomotors by BMSCs cocultured with different NMDS and the labeling efficiency of nanomotors on BMSCs. (F) Schematic illustration of the NMDS recruiting BMSCs. (G) Crystal violet staining of BMSCs migrated from the upper chambers. (H) Quantitative number of migrated BMSCs based on the crystal violet staining (n = 20, ^*^p < 0.05 and ^**^ ^*^p < 0.001). (I) GAG content synthesized by BMSCs cocultured with the NMDS in complete and chondrogenic medium under the 3D MF actuation (75 mT, [2, 1] Hz, 10 min/day) (n = 3, ^*^p < 0.05, ^*^ ^*^p < 0.01, and ^**^ ^*^p < 0.001). (J) Schematic illustration of the endogenous BMSCs recruitment evaluation in vivo. (K, L) Immunofluorescence staining of BMSCs with specific markers CD90 and CD105 in the cartilage defects after the NMDS implantation for 7 days and 14 days. (M) The relative double positive cell percentage of BMSCs at the cartilage defects (n = 4, ^**^ ^*^p < 0.001).

To address the rapid clearance of nanomotors in the articular cavity that hinders the persistent dynamic mechanical modulation on endogenous BMSCs, thermosensitive hydrogels with injectability are advocated as the local delivery system for nanomotors to ensure sustained and controlled release at the injury sites [43]. In this study, a thermosensitive hydrogel (TGel) composed of an arthrosis‐beneficial drug, sodium hyaluronate, and an FDA‐approved thermosensitive polymer, Pluronic F‐127, was adopted to load the E7@RFNMs, forming the nanomotor delivery system (NMDS). The detailed characterizations, rheological evaluation, cytocompatibility, and hemocompatibility of the NMDS were described and shown in the Figure S11–S14. The physically cross‐linked NMDS could be completely dissociated within 16 days, enabling the continuous release of nanomotors (Figure 6A) while retaining their original morphology integrity (Figure S15A and Figure 6B). The sustained release of nanomotors allowed them to label BMSCs in cartilage defects, delivering intracellular magneto‐mechanical stimulation durably in response to the external 3D MF. Besides, the non‐covalently adsorbed E7 peptide on nanomotor surfaces exhibited a cumulative release profile of 58.8% within 16 days (Figure 6C). The slowly released fraction recruited endogenous BMSCs, while the nanomotor‐bound fraction mediated specific cellular uptake, paving the way for intracellular dynamic magneto‐mechanical stimulation. Prussian blue staining and quantitative results from inductively coupled plasma‐optical emission spectroscopy demonstrated effective, concentration‐dependent labeling of BMSCs by the released nanomotors from the NMDS (Figure 6D,E). An optimized nanomotor loading of 10.4 pg/cell in BMSCs was achieved with the NMDS‐80 µg/mL formulation (Figure 6E), a level comparable to direct incubation methods (Figure S4B, 9.5 pg/cell at the feed concentration of 10 µg/mL). Hence, NMDS‐80 µg/mL was selected in the following experiments unless otherwise stated.

Employing a Transwell chamber to imitate the relative position between the implanted NMDS and endogenous BMSCs, BMSCs were seeded into the upper chamber and assumed to be recruited by the released E7 peptide to migrate into the lower chamber (Figure 6F). The E7@RFNM‐loaded NMDS demonstrated significant recruitment ability to BMSCs, attracting a larger number of BMSCs compared to E7 peptide‐free control groups (Figure 6G,H). Moreover, 3D MF actuation could enhance the migration of E7@RFNM‐labeled BMSCs, as demonstrated by the accelerated wound healing in the 3D MF‐actuated NMDS group with a cell scratch assay (Figure S15B,C). Consistent with the above results (Figure 4E,F), the 3D MF‐actuated NMDS also potently promoted the synthesis of GAG in BMSCs compared to other groups both in complete and chondrogenic media (Figure 6I).

To further evaluate the impact of the NMDS on the recruitment and mobilization of endogenous BMSCs in vivo, the NMDS was implanted in the rat cartilage defects post microfracture surgery, and the tissue was harvested after 7 and 14 days (Figure 6J). The immunofluorescence staining revealed that the CD90/CD105 double‐positive signals in E7@RFNM‐loaded NMDS groups obviously exceeded those in E7 peptide‐free control groups (Ctrl and PDA@RFNM DS) after 7 days and 14 days of implantation (Figure 6K,L). The quantitative CD90/CD105 double‐positive cell percentage also yielded consistent results that E7@RFNM‐loaded NMDS significantly increased the enrichment of endogenous BMSCs at cartilage defects compared to E7 peptide‐free control groups (Figure 6M). From the Prussian blue & Nuclear fast red (PB & NR) staining of cartilage defects, the NMDS groups showed dispersed blue signal after 7 days and 14 days of implantation compared to the Ctrl group, indicating the sustained presence of nanomotors at defects (Figure S16). These findings suggested that the NMDS was reliable for enhanced endogenous BMSCs mobilization towards the defect area and persistent dynamic magneto‐mechanical stimulation on BMSCs. In addition, with the antioxidative properties inherited from nanomotors, the NMDS was demonstrated to endow BMSCs with enhanced resistance against oxidative damage by reducing ROS levels and suppressing the inflammatory microenvironment (Figure S17).

**FIGURE 7:**
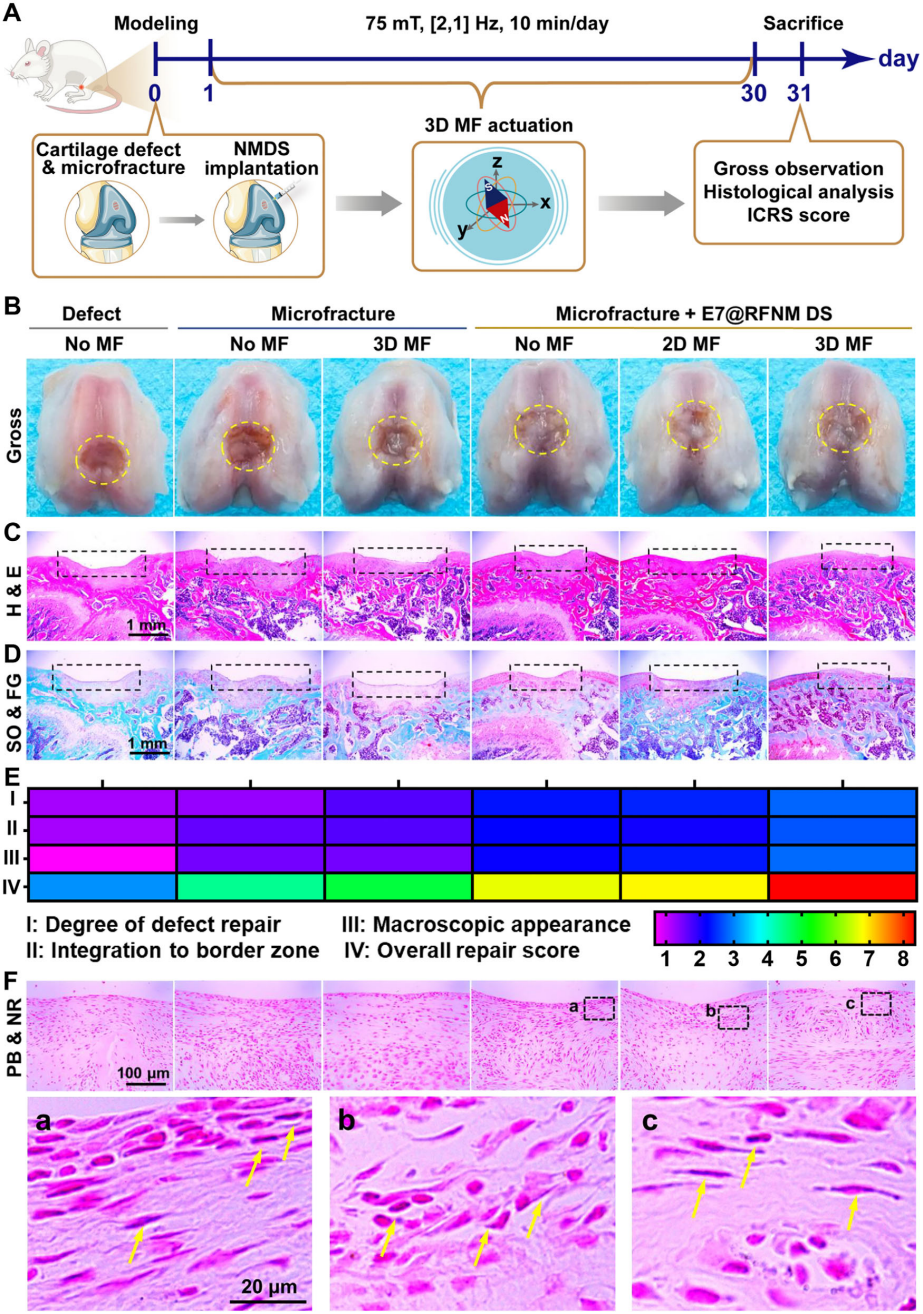
In situ magneto‐mechanical modulation of endogenous BMSCs for enhanced cartilage repair. (A) Illustration of the modeling and treatment process, as well as the therapeutic effect evaluation. (B) Representative macroscopic photographs of the joint samples after one month of treatment. The yellow circles represented the region of the cartilage defect. (C, D) H & E staining and SO & FG staining of femoral sample sections. The black rectangles represented the repaired tissue in the cartilage defect. (E) Cartilage repair assessment by the ICRS scoring system. (F) PB & NR staining of femoral sample sections. The enlarged images (a‐c) represented the positive regions of Prussian blue.

Encouraged by the capabilities in BMSC recruitment, protection, and modulation of chondrogenic commitment, the 3D MF‐actuated NMDS was employed to seek enhanced therapeutic effects on cartilage defects in combination with the microfracture technique through intervening in the cartilage regeneration process dominated by endogenous BMSCs. The surgery and treatment procedures were illustrated in Figure 7A, and no obvious complications related to implantation were observed throughout the whole process. After one month of treatment, the gross appearance of the cartilage sample in the Defect group showed few of newborn tissue, suggesting the limited self‐repair of cartilage. Although the newborn tissues in Microfracture groups (with no MF or 3D MF actuation) showed slight improvement compared with those in the Defect group, the defects were still not completely filled, confirming the inadequate cartilage repair of microfracture surgery without effective intervention. In contrast, the Microfracture + E7@RFNM DS groups (with no MF, 2D MF or 3D MF actuation) exhibited relatively more newborn tissue filling, and especially, the Microfracture + E7@RFNM DS + 3D MF group was almost entirely filled (Figure 7B).

Histochemical analysis showed a consistent trend with the gross observation. Form the Hematoxylin & Eosin (H & E) staining, the neocartilage in the Microfracture + E7@RFNM DS + 3D MF group was more similar to the original cartilage in the sham group than other groups that lacked the normal orientation structure of cartilage (Figure 7C and Figure S18). From the Safranine O & Fast green (SO & FG) staining, the neocartilage in E7@RFNM‐free groups showed weak red signals, indicating a trend towards the formation of fibrocartilage instead of hyaline cartilage. By contrast, the evident red staining of neocartilage in E7@RFNM‐loaded groups demonstrated the presence of hyaline cartilage in agreement with the superior BMSC recruitment and protection provided by the E7@RFNM DS (Figure 7D). In particular, the Microfracture + E7@RFNM DS + 3D MF group exhibited the best repair outcomes compared with other groups, underscoring the superiority of the 3D MF‐actuated E7@RFNM DS in microfracture enhancement.

In addition, the International Cartilage Repair Society (ICRS) cartilage repair assessment also revealed that the Microfracture + E7@RFNM DS + 3D MF group achieved the highest overall repair score among all groups (Figure 7E). From the PB & NR staining, the blue signal was observed in E7@RFNM‐loaded groups, indicating that the nanomotors were retained in the cartilage defect region (Figure 7F). After the microfracture surgery, the resident nanomotors recruited and targeted endogenous BMSCs, and further modulated the chondrogenesis under the actuation of the 3D MF. These results suggested that the dynamic magneto‐mechanical stimulation mediated by the 3D MF coupling with the E7@RFNMs could serve as a “microfracture plus” technique [44, 45, 46] for enhanced cartilage repair by optimizing the regeneration process to overcome the clinical limitations of conventional microfracture surgery.

## 3 Discussion

In this study, we established a high‐fidelity magneto‐mechanical actuation platform for wireless mechanical‐stimulated cell fate programming by remotely manipulating organelle‐level mechanics. By coupling intracellular nanomotors with a rotating fluctuating 3D MF, the platform successfully orchestrated a robust in situ chondrogenic commitment of endogenous MSCs and achieved enhanced cartilage repair in a rat knee articular cartilage defect model. Unlike conventional approaches that rely on passive bio‐scaffolds or extracellular mechanical loading, this strategy utilized the trifunctional nanomotors as active intracellular engines, providing both biochemical protection and biomechanical modulation, and thereby overcame the critical limitations of endogenous BMSCs within the hostile microenvironment characterized by oxidative stress and aberrant mechanical cues. Our findings deepened the understanding of the effects of dynamic mechanical stimulation on the regulation of MSC fate and functions, which is required for more effective and efficient stem cell‐based therapies aimed at the regeneration of load‐bearing tissues.

The key to this platform lies in the high‐efficiency physical actuation mode. The nanomotors performed a unique trans‐planar rotational‐bouncing motion in cellular lysosomes under the actuation of 3D MF, significantly outperforming conventional 2D planar rotation mode. From a physical perspective, the superiority of this 3D actuation over conventional 2D modes is rooted in fluid dynamics at the microscale. In the viscous environment of the lysosome, simple 2D planar rotation often encounters the “scallop theorem” limitation, generating minimal net disturbance. In contrast, unlocking of the third dimension of magnetic actuation breaks this symmetry, creating a high‐shear fluid vortex. Our theoretical simulation confirmed that this 3D actuation generates significantly higher fluid velocity gradients and shear stresses than 2D mode, translating into more potent magneto‐mechanical outputs, as evidenced by the force‐sensitive DNA probe assay requiring lower frequencies to achieve equivalent mechanical melt. This explained why our platform could trigger robust chondrogenic gene expression at lower MF frequencies and nanomotor dosages. This enhanced magnetic‐mechanical conversion efficiency is critical for clinical translation, mitigating biosafety concerns associated with high particle loads or intense magnetic fields.

Our results established a clear magnetic torque‐dependent effect for programming the chondrogenic fate of BMSCs. By systematically tuning the frequency, mode, and duration of the 3D MF, we identified an optimal parameter set (10 µg/mL of nanomotors, [2, 1] Hz of 3D MF, 10 min/day of duration) that maximally upregulated chondrogenic genes and enhanced the synthesis of cartilage‐specific matrix proteins. This programmable control highlights the platform's versatility and potential for personalized mechano‐therapy. Mechanistically, we identified the lysosomal mechanically‐gated actin cytoskeletal remodeling as the primary regulator of this intracellular mechanical programming for cell fate. Lysosomes have been viewed not only as a terminal degradative compartment but also as a central signaling nexus that orchestrates cellular homeostasis, with profound implications for cytoskeletal architecture and dynamics [47, 48]. We demonstrated that the dynamic magneto‐mechanical stimulation within lysosomes triggered rapid LMP and subsequent cytosolic translocation of CathD. CathD exerted its cofilin phosphatase function to mediate the reorganization of actin cytoskeleton, which is a well‐known mechanotransduction switch of MSC chondrogenesis [36, 49, 50, 51]. We found that the pharmacological disruption of actin by CytoD further potentiated the magneto‐mechanical induced upregulation of chondrogenic genes, whereas stabilizing actin with Jasp inhibited this promoting effect. It strongly supports that cytoskeletal remodeling is a key central transducer activated by magneto‐mechanical actuation. These findings align with reports linking cytoskeletal disruption to enhanced Sox9 expression and chondrogenesis [37, 52, 53, 54], confirming that the lysosomal mechanical perturbation effectively taps into established mechanotransduction signaling cascades to direct differentiation.

To translate this cellular‐level programming into tissue‐level regeneration, we engineered an intra‐articularly injectable thermosensitive delivery system, addressing the challenge of sustained nanomotor retention in the articular cavity. Through the gradual release of nanomotors and E7 peptide, the NMDS created a “recruitment‐protection‐actuation” system, enabling effective recruitment and label of endogenous BMSCs, protection of BMSCs against oxidative damage, and prolonged intracellular mechanical stimulation. Our results are consistent with prior studies highlighting the synergy between biochemical microenvironmental regulation and biophysical mechanical modulation [55, 56]. Importantly, 3D MF actuated NMDS combined with the microfracture technique significantly enhanced hyaline cartilage repair in vivo, surpassing the limited outcomes of microfracture surgery alone. These results suggest that this system may be considered as a “microfracture plus” strategy, offering clinical translational potential [57]. This study affirms the potential effects of dynamic mechanical stimulation on the regulation of MSC fate and functions, and further extends the concept by demonstrating intracellular, non‐contact manipulation as an effective alternative for cartilage regeneration.

While magnetic actuation or stimulation has been broadly explored for regeneration of various tissues, including bone [58], muscle tendon [59], and particularly cartilage [60], our 3D magneto‐mechanical stimulation platform introduces a distinct paradigm shift compared to existing approaches. Conventional magnetic actuation for tissue engineering primarily relies on macroscopic magnetic scaffolds. These macroscopic approaches are typically realized by indirectly transferring bulk mechanical strain to seeded cells through tunable mechanical properties [60, 61, 62], or by guiding the directed alignment of magnetically labeled cells to construct biomimetic tissue architectures [58, 59, 63]. However, for clinical translation, these macroscopic scaffolds face the formidable challenge of carefully balancing mechanical properties, degradation kinetics, and biocompatibility. Alternatively, while emerging cell‐based magnetic microrobots [64] or microcarriers [65, 66] offer high spatiotemporal precision, they are predominantly utilized as vehicles for the targeted delivery of stem cells or therapeutics, making it difficult to achieve direct and precise mechanical intervention on the stem cells themselves. In contrast, our platform utilizes magnetic nanomotors to directly perform spatiotemporally controllable mechanical modulation at the subcellular level by specifically targeting lysosomes. By engineering the 3D rotating fluctuating magnetic field, the designed system overcomes the hydrodynamic limitations inherent to conventional planar motion within highly viscous intracellular environments, thereby amplifying the intracellular fluid vortices and shear stress by the unique rotational‐bouncing motion of magnetic nanomotors. Furthermore, unlike conventional strategies that primarily focus on pre‐conditioning exogenous stem cells in vitro prior to transplantation, our platform is tailored for the complex in vivo microenvironment, utilizing a clinically approved injectable hydrogel for minimally invasive intra‐articular delivery. By integrating antioxidative properties and MSC‐targeting affinity, the nanomotors concurrently neutralize local oxidative stress and actively recruit microfracture‐liberated endogenous BMSCs, achieving an in situ multimodal programming of cell fate that transcends pure mechanical stimulation. However, it is critical to acknowledge the potential limitations of this strategy. The in vivo degradation profiles, long‐term metabolism, and comprehensive biosafety of the internalized magnetic nanomotors remain latent concerns that necessitate systematic and extensive evaluation prior to broader clinical applications.

Despite the rat cartilage regeneration model providing valuable proof‐of‐concept evidence, this magneto‐mechanical actuation platform holds broad potential for directing other lineage specifications or modulating cell behaviors in diverse tissue engineering applications. The current magnetic field setup may require scaling and optimization to be adapted for a wider range of biomechanical applications. Future work will focus on developing more compact, clinically applicable magnetic actuation devices for these wireless control strategies. Besides, there may be other upstream sensors that mediate the actin remodeling to be systematically investigated. Because magneto‐mechanical stimulation‐triggered LMP is not a single event, and the leakage of lysosomal contents is not specific. Finally, the long‐term biodegradation profile of nanomotors in vivo and comprehensive toxicology over extended periods requires systematic assessment prior to clinical applications.

## 4 Conclusion

This study presented a programmable intracellular magneto‐mechanical stimulation platform capable of orchestrating the in situ chondrogenic commitment of endogenous BMSCs through high‐fidelity physical cues. The cornerstone of this strategy is the 3D MF actuated nanomotors to execute a unique trans‐planar rotational‐bouncing motion inside cellular lysosome. In vitro experiments confirmed that this multimodal motion overcame the hydrodynamic limitations of conventional planar motion, generating amplified intracellular dynamic mechanical stimulation for enhanced chondrogenic differentiation as evidenced by upregulated chondrogenic gene expression and cartilage‐specific matrix protein synthesis. The underlying mechanism was identified as lysosomal mechanical perturbation induced actin cytoskeleton remodeling and downstream signaling pathway activation. The magnetic nanomotors were designed to integrate antioxidative activity and MSC‐targeting capability. Following intra‐articular injection with the delivery system, this platform enabled recruitment and targeting of endogenous BMSCs, protecting them from oxidative damage and mechanical programming their chondrogenic fate. A proof‐of‐concept in vivo model demonstrated that the platform promoted hyaline cartilage repair when combined with the microfracture surgery under the remote actuation of the 3D MF. This platform provided a versatile, wireless toolkit for the “mechanical programming” of cell functions, opening a new avenue for bridging nanorobotics and regenerative medicine.

## 5 Experimental Methods

### 5.1 Synthesis of Rhein@Fe_3_O_4_ Nanomotors (RFNMs)

The RFNMs were synthesized via the typical solvothermal method with a slight modification. Briefly, rhein (30 mg) was dissolved in ethylene glycol (30 mL) containing polyethyleneimine (300 mg), followed by the addition of polyvinylpyrrolidone (PVP, 1.0 g). After complete dissolution, FeCl_3_·6H_2_O (0.68 g) was added into the above solution to form the PVP‐rhein‐Fe^3+^ complex with vigorous stirring. Then, sodium acetate (3 g) was added to the mixture and further stirred uniformly. The mixture was sealed in a Teflon‐lined stainless‐steel autoclave (50 mL capacity). Subsequently, the autoclave was heated to and maintained at 200°C for 12 h and then allowed to cool to room temperature. The black product was collected, washed three times with ethanol, and finally stored at 4°C.

### 5.2 Preparation of E7 Peptide‐Modified RFNMs (E7@RFNMs)

Polydopamine (PDA) was first coated onto the RFNMs to provide adsorption sites. Dopamine hydrochloride (1 mg) was dissolved in Tris‐HCl (pH = 8.5, 10 mL), followed by the addition of RFNMs (10 mg). The solution was stirred continuously at room temperature for 12 h to form PDA@RFNMs through the oxidative polymerization of dopamine. The PDA@RFNMs were then repeatedly rinsed with deionized water for further experiments. Subsequently, the PDA@RFNMs were activated by dispersing in PBS containing 0.15 M NaCl. The activated PDA@RFNMs (10 mg) were dispersed in Tris buffer (10 mL) containing E7 peptide (2 mg), and then stirred continuously at 4°C for 24 h to form E7@RFNMs. Finally, the product was centrifugated (15000 rpm, 10 min), and the supernatant was collected for peptide quantification. The E7@RFNMs were further washed thoroughly with deionized water.

### 5.3 Characterizations of E7@RFNMs

A transmission electron microscope (TEM, Jeol JEM‐1230, Japan) was employed to observe the morphology of nanomotors. The hydrodynamic diameters and zeta potentials of nanomotors were measured using a dynamic light scattering system (DLS, Malvern Zetasizer Nano‐90, UK). Fourier transform infrared (FTIR) spectra of nanomotors were obtained with an infrared spectrometer (Thermo Fisher Scientific Nicolet iS20, USA) in the range of 400 to 4000 cm^−1^ with a resolution of 4 cm^−1^ by the KBr pellet technique. A BCA protein assay kit (Beyotime, China) was used to quantify the peptide content on nanomotors. The x‐ray diffraction (XRD) patterns of nanomotors were obtained from an x‐ray powder diffractometer (Brucker D8 ADVANCE, Germany) with the monochromatic x‐ray of λ = 1.5406 Å. X‐ray photoelectron spectroscopy (XPS) measurements of nanomotors were performed on an x‐ray photoelectron spectroscope (Thermo ESCALAB 250 XI, USA) under an ultra‐high vacuum less than 5 × 10^−8^ Torr. The magnetic hysteresis curves of nanomotors were measured with a vibrating sample magnetometer (Lake Shore 8604, USA) operating at a magnetic field strength of ± 2T and 300 K.

### 5.4 Antioxidative Activity Evaluation of Nanomotors

The antioxidative properties of nanomotors were evaluated by the scavenging rates of the model radical DPPH·. DPPH· stock solution (50 µg/mL) was prepared by dissolving the DPPH powder in ethanol. Then, 0.5 mL of radical solution was mixed with twofold serial dilution of nanomotor solutions (1 mL) to achieve final concentrations of 80, 40, 20, 10, 5 µg/mL as [Fe]. After incubation at 37°C for 30 min and centrifugation, the absorbance of the supernatant was recorded at the wavelength of 519 nm using a UV–vis spectrometer (Agilent Cary 60, USA). The radical scavenging rates were calculated using the following equation:

(1) $$\begin{array}{ccc} \mathrm{Radical}\;\mathrm{scavenging}\;\mathrm{rate}\;\left(\%\right) & = & \left({\mathrm{Abs}}_{\mathrm{cont}\mathrm{rol}}-\mathrm{Ab}s_{\mathrm{sample}}\right)/\mathrm{Ab}s_{\mathrm{control}}\\ & & \times 100 \end{array}$$

where Abs_control_ is the absorbance of pure radical solution without nanomotors, and Abs_sample_ is the absorbance of radical solution after reaction with nanomotors.

### 5.5 Configurations of the Rotating Fluctuating 3D MF

A self‐developed magnetic actuation system composed of solenoid coils and rotatable Halbach permanent magnet arrays was constructed. The system can independently tune the current configuration of solenoid coils to output an axial EMF with oscillatory mode, and independently adjust the rotational speed of magnet arrays to output a radial PMF with rotational mode. When synchronously controlling the magnet arrays and coil current, the rotating fluctuating 3D MF was generated through coupling the oscillatory EMF and rotational PMF enabling contactless five‐degree‐of‐freedom manipulation of magnetic nanomotors. The frequency ([x,y] Hz) of the 3D MF was expressed as a combination of the EMF frequency (x Hz) and the PMF frequency (y Hz). The magnetic field strength was measured at 75 mT. Likewise, another self‐developed minitype magnetic actuation system, which is composed of eight solenoid coils and pure iron cores, was used to display and record the locomotion behaviors of nanomotors under a microscope (Olympus IX 71, Japan). This system can generate equivalent 3D MF through real‐time adjustment of current configurations in its eight‐coil array.

### 5.6 Magneto‐Mechanical Output Detection Using Force‐Sensitive DNA Probes

The DNA probe with a melting threshold of 12 pN was composed of a BHQ2‐conjugated quencher strand containing an azido end (5’‐/BHQ‐2/GGCCCGCAGCGACCACCC/N_3_/‐3’) and a Cy3‐labeled fluorescence strand containing a biotin end (5’‐/Biotin/TTTTTTTTTTGGGTGGTCGCTGCGGGCC/Cy3/‐3’). The quencher strands (0.15 nm) and the fluorescence strands (0.1 nM) were mixed in TAE buffer (1× TAE containing 20 mm MgCl_2_), and then the mixture was added onto the streptavidin‐modified glasses for 30 min at room temperature. Subsequently, the dibenzocyclooctyne‐modified nanomotors were added onto the glasses for 60 min at 37°C to link with the quencher strand through the click chemistry. After washing with PBS, the glasses were exposed to different frequencies of 2D MF and 3D MF for 10 min. The fluorescence images were captured on a confocal laser scanning microscope (CLSM, LSM880, Zeiss, Germany). The glass only modified with the fluorescence strands served as the positive control, while those only modified with the quencher strands served as the negative control.

### 5.7 Cell Acquisition and Culture

Bone marrow mesenchymal stem cells (BMSCs, Cat# RASMX‐01001, RRID: CVCL_A9JU) from Sprague‐Dawley (SD) rats (RRID: MGI: 5651135) were purchased from Cyagen Biosciences and cultured with a tailor‐made complete medium (RAXMX‐90011, Cyagen Biosciences, China) at 37°C with 5% CO_2_ atmosphere. Cells were cultured until reaching 80% confluence and subsequently sub‐cultured at a 1:2 split ratio. Cells from passages 3 to 5 were utilized for cellular experiments.

### 5.8 Evaluation of Nanomotors Uptake by BMSCs

BMSCs were cocultured with different concentrations of PDA@RFNMs or E7@RFNMs for 24 h. Next, the cells were washed with PBS and fixed with paraformaldehyde solution (4%) for 20 min. The cells were then stained with the Prussian blue iron stain kit (with nuclear fast red, G1422, Solarbio, China) according to the manufacturer's instructions, and the staining images were captured by an EVOS FL Auto cell imaging system (Life technologies, Thermo Fisher Scientific, USA). Besides, the cells after cocultured with different concentrations of PDA@RFNMs or E7@RFNMs for 24 h were washed with PBS, resuspended, and counted. Then, the cells were digested in chloroazotic acid for measuring Fe content using an inductively coupled plasma‐optical emission spectrometer (Thermo iCAP 7600 ICP‐OES, USA).

### 5.9 Localization Analysis of E7@RFNMs in BMSCs

BMSCs were cocultured with 20 µg/mL of FITC‐E7@RFNMs for 24 h. Then, the cells were washed with PBS and stained with the Lyso‐Tracker Red (C1046, Beyotime, China) and Hoechst (33258, Beyotime, China), respectively, according to the manufacturer's instructions. The staining images were captured on a CLSM (LSM880, Zeiss, Germany). The colocalization between nanomotors and lysosomes was analyzed by the ImageJ software.

### 5.10 Cell Viability and Proliferation of Nanomotor‐Labeled BMSCs

BMSCs were cocultured with different concentrations of PDA@RFNMs or E7@RFNMs for 1, 3, 5, and 7 days, respectively. Inoculated wells without nanomotors were used as a control. For the magneto‐mechanical stimulation, the cells were exposed to the 3D MF with different frequencies for 10 min/day. At a specific time point, the cells were incubated with CCK‐8/DMEM solution (10%, v/v) for 2 h, and the supernatant was withdrawn to measure the optical density (OD) at the wavelength of 450 nm on a microplate reader (Tecan, Infinite 200 Pro, Switzerland). The relative cell viability/percentage was calculated by taking the OD of the control group in day 1 as 100%. Next, the cells were fixed with paraformaldehyde solution (4%) for 20 min, and stained with crystal violet solution (0.1%) for 30 min. The staining images were captured by a cell imaging system.

### 5.11 Evaluation of Chondrogenic Gene Expression in E7@RFNM‐Labeled MSCs

BMSCs were cocultured with E7@RFNMs for 24 h. Then, the cells suffered the magneto‐mechanical stimulation by exposure to the 3D MF with different conditions, which were changed by adjusting the nanomotor concentrations, MF frequencies, duration time per day, and number of days. Besides, the effect of culture medium was also investigated using the complete medium and the chondrogenic medium (complete medium supplemented with 50 µg/mL ascorbic acid, 50 µg/mL Insulin‐Transferrin‐Selenite premix, 10 nM dexamethasone, and 10 ng/mL TGF‐β3). Next, the expression of chondrogenic genes (*Col2a1*, *Acan*, and *Sox9*) was detected with a real‐time quantitative reverse transcription polymerase chain reaction (RT‐qPCR) assay. The total RNA was isolated using TRIzol reagent (Invitrogen 9994001, Thermo Fisher Scientific, USA), and cDNA was synthesized using a reverse transcription kit (ReverTra Ace qRCR RT Master Mix, FSQ‐201, TOYOBO, Japan) according to the manufacturer's instructions. Then, cDNA was mixed with primers and the 2X SG Fast qPCR Master Mix (BBI, B639272, China), and the PCR was performed on the QuantStudio 7 Flex real‐time PCR system (ABI, USA). The expression of target genes was analyzed using the 2^−ΔΔCt^ method, taking the glyceraldehyde‐3‐phosphate dehydrogenase (GAPDH) as the reference gene. The sequences of primer pairs were shown in the Table S1.

### 5.12 Qualitative Evaluation of GAG by Alcian Blue Staining

BMSCs were cocultured with 10 µg/mL of E7@RFNMs in complete medium or chondrogenic medium for 24 h, and exposed to the 3D MF (75 mT, [2, 1] Hz) or not for another 7 days (10 min/day). Then, the cells were washed with PBS and fixed with paraformaldehyde solution (4%) for 20 min. The cells were then stained with Alcian blue reagent (Cyagen Biosciences, ALCB‐10001, China) according to the manufacturer's instructions, and the staining images were captured by a cell imaging system. The optical density of images was analyzed by the ImageJ software.

### 5.13 Immunofluorescent Staining of COL II

BMSCs were treated as in the above section. After fixed, the cells were permeabilized with Triton‐X 100 (0.5%) at 4°C for 30 min, and blocked the unspecific binding of antibodies by incubating with BSA solution (1%). Subsequently, the cells were incubated with a primary antibody (Collagen type II polyclonal antibody, Proteintech Cat# 28459‐1‐AP, RRID: AB_2881147) at 4°C overnight and a second antibody (Goat anti‐rabbit IgG H&L, Alexa Fluor 488, Abcam Cat# ab150081, RRID: AB_2734747) for 2 h at room temperature, respectively. Then, cell nuclei were stained with the 4’,6‐diamidino‐2‐phenylindole (DAPI, Beyotime, China). Finally, the fluorescent images were captured on a CLSM (Nikon, TI2‐E+AX R, Japan). The fluorescence intensity of images was analyzed by the ImageJ software.

### 5.14 COL II expression Evaluation by Western‐Blot Analysis

BMSCs were treated as in the above section. After washing with PBS, the cells were lysed with a cell lysis buffer containing protease inhibitor (Beyotime, P1005, China) and centrifuged. The supernatant (protein lysate) was collected to measure the protein concentrations using a BCA protein assay kit. Then, sodium dodecyl sulfate‐polyacrylamide gel electrophoresis was used to separate the lysate. The separated lysate was transferred to polyvinylidene fluoride membranes. The membranes were blocked in a quick‐blocking buffer (Beyotime, P0235, China) at room temperature for 15 min, and incubated in the primary antibody dilutions at 4 °C overnight. The membranes were washed thrice with PBS containing Tween‐20 (PBST, Sinopharm, China), and incubated in the second antibody dilutions at room temperature for 2 h. After being washed thrice with PBST again, they were developed with an Omni‐ECL UltraSensitive Chemiluminescent Detection Kit (Epizyme, SQ201, China) for 1 min and imaged using a Tanon 5200CE imaging system. The primary antibodies and dilutions used were as follows: GAPDH antibody (1:2000, Santa Cruz Biotechnology Cat# sc‐32233, RRID: AB_627679), COL II polyclonal antibody (1:1000, Affinity Biosciences Cat# AF0135, RRID: AB_2833318). The secondary antibodies and dilutions used were as follows: anti‐rabbit IgG, HRP‐linked antibody (1:5000, Cell Signaling Technology Cat# 7074, RRID: AB_2099233) or anti‐mouse IgG, HRP‐linked antibody (1:5000, Cell Signaling Technology Cat# 7076, RRID: AB_330924).

### 5.15 Actin Cytoskeleton Characterization and p‐Cofilin Level Evaluation of BMSCs

BMSCs were cocultured with 10 µg/mL of E7@RFNMs for 24 h, and then exposed to the 3D MF (75 mT, [2, 1] Hz) for 10 min. At the predetermined time points after the magneto‐mechanical stimulation, the cells were fixed and permeabilized with Triton‐X 100 (0.5%). Then, the cells were stained with Actin‐Tracker Green‐488 (Beyotime, C2201S, China) and DAPI. Finally, the images were obtained by a CLSM (Nikon, TI2‐E+AX R, Japan). BMSCs with identical treatment were lysed to collect the total protein, and then a general Western blot procedure was conducted to analyze the p‐cofilin level. The primary antibodies and dilutions used were as follows: Phospho‐cofilin monoclonal antibody (1:1000, Cell Signaling Technology Cat# 3313, RRID: AB_2080597), cofilin monoclonal antibody (1:1000, Cell Signaling Technology Cat# 5175, RRID: AB_10622000). The secondary antibodies and dilutions used were as follows: anti‐rabbit IgG, HRP‐linked antibody (1:5000, Cell Signaling Technology Cat# 7074, RRID: AB_2099233) or anti‐mouse IgG, HRP‐linked antibody (1:5000, Cell Signaling Technology Cat# 7076, RRID: AB_330924).

### 5.16 Effect Evaluation of Actin Disruption on Chondrogenic Gene Expression

BMSCs were cocultured with 10 µg/mL of E7@RFNMs for 24 h, and then exposed to the 3D MF (75 mT, [2, 1] Hz) or not for 3 days (10 min/day). Before the magneto‐mechanical stimulation every day, the cells were treated with 2 µm CytoD (Aladdin, China) or 0.2 µm Jasp (Abcam, UK) for 1 h. Finally, the cells were collected for detecting the expression of chondrogenic genes (*Col2a1*, *Acan*, *Sox9*) by RT‐qPCR.

### 5.17 Characterization of Lysosomal Membrane Permeabilization and Lysosome Movement

For lysosomal membrane permeabilization characterization, BMSCs were transfected with enhanced green fluorescent protein‐tagged galectin‐3 (EGFP‐Gal3, RRID: Addgene_73080) plasmids using jetPRIME (Polyplus‐Sartorius, USA) as transfection reagents according to the manufacturer's instructions. Then, the cells were cocultured with 10 µg/mL of E7@RFNMs for 24 h and exposed to the 3D MF (75 mT, [2, 1] Hz) for 10 min. After fixed, the cells were stained with the Lyso‐Tracker Red and DAPI, respectively. The staining images were captured on a CLSM (Nikon, TI2‐E+AX R, Japan). The Gal3 puncta per cell were analyzed by the ImageJ software. For lysosome movement observation, BMSCs were cocultured with 10 µg/mL of E7@RFNMs for 24 h, and stained with the Lyso‐Tracker Red and Hoechst. Then, the cells were exposed to the 3D MF, and simultaneously, the time‐lapse images were captured on a CLSM (Nikon, TI2‐E+AX R, Japan). The tracking and displacement of lysosome movement were by the ImageJ software.

### 5.18 Characterization of CathD Leakage by Immunofluorescent Staining

BMSCs were cocultured with 10 µg/mL of E7@RFNMs for 24 h, and then exposed to the 3D MF (75 mT, [2, 1] Hz) for 10 min. After fixed, permeabilized and blocked, the cells were incubated with primary antibodies Cathepsin D monoclonal antibody (1: 1000, Abcam Cat# ab75852, RRID: AB_1523267) and LAMP1 antibody (1: 500, Proteintech Cat# 32435‐1‐AP, RRID: AB_3742610) at 4°C overnight and second antibodies Goat anti‐rabbit IgG H&L (Alexa Fluor 488, Abcam Cat# ab150081, RRID: AB_2734747) and Goat anti‐mouse IgG H&L (Alexa Fluor 555, Abcam Cat# ab150114, RRID: AB_2687594) for 2 h at room temperature, respectively. Then, cell nuclei were stained with DAPI. Finally, the fluorescent images were captured on a CLSM (Nikon, TI2‐E+AX R, Japan). The Manders’ coefficient of images was analyzed by the ImageJ software.

### 5.19 In Vitro Release Evaluation of Nanomotors and E7 Peptide from the NMDS

The nanomotors and E7 peptide release profiles of the NMDS were measured using a standard procedure. Briefly, 3 mL of NMDS precursor containing 80 µg/mL of FITC‐E7@RFNMs was injected into an empty vial weighed as M_v_, incubated at 37°C to gelation, and accurately weighed as M_0_. Then, 1 mL of PBS was added to the vial. At predetermined time points, the vial was weighed as M_t_ after the liquid from the upper layer had been carefully removed, and another 1 mL of fresh PBS was added. After the removed liquid was centrifuged, the precipitate was digested in chloroazotic acid for measuring Fe content by ICP‐OES, and the supernatant was used to record the fluorescence intensity at Ex. 488 nm/Em. 519 nm by a microplate reader. The concentration of released E7 peptide was determined from a standard curve of FITC‐E7 peptide. The mass remaining of NMDS, and the cumulative release of nanomotors and E7 peptide were calculated using the following equations:

(2) $$\mathrm{Mass}\;\mathrm{remaining}\;\left(\%\right)=\left(M_{t}-M_{v}\right)/\left(M_{0}-M_{v}\right)\times 100$$

(3) $$\mathrm{Cumulative}\;\mathrm{release}\;\left(\%\right)=\left(C_{1}+C_{2}+\text{…}+C_{i}\right)/C_{0}\times 100$$

where C_i_ represents the content of Fe or E7 peptide released at day i, and C_0_ represents the initial content of Fe or E7 peptide.

### 5.20 Evaluation of NMDS‐Released Nanomotor Uptake by BMSCs

After BMSCs were incubated for 24 h, different concentrations of E7@RFNMs loaded NMDS were injected to cover the cells. After incubation for another 24 h, the cells were fixed for Prussian blue staining or collected for measuring Fe content by ICP‐OES.

### 5.21 Recruitment and Migration Evaluation of BMSCs on the NMDS

The ability of NMDS to recruit BMSCs was tested by the Transwell assay. BMSCs were seeded in the upper chamber of the Transwell plate (PET membrane, 8.0 µm), and the NMDS was injected into the bottom chamber. The medium used in this assay was serum‐free. After 24 h of coculture, the migrated cells were fixed with paraformaldehyde solution (4%), stained with crystal violet (0.1%), and finally counted using the ImageJ software after imaging by a cell imaging system. The migration ability of BMSCs on the NMDS was tested by the scratch assay. After BMSCs were cocultured with the NMDS for 24 h, wounds were created by scraping the bottom with 1 mL pipette tips. Then, the cells were exposed to the 3D MF (75 mT, [2, 1] Hz, 10 min) or not. After incubation for another 24 h, the cells were fixed with paraformaldehyde solution (4%), and stained with crystal violet (0.1%). The wound areas were observed by a cell imaging system and analyzed using the ImageJ software.

### 5.22 Quantitative Evaluation of GAG by DMMB Colorimetry

BMSCs were cocultured with the NMDS in complete medium or chondrogenic medium for 24 h, and then exposed to the 3D MF (75 mT, [2, 1] Hz) or not for another 7 days (10 min/day). Next, the total protein of the cells was collected with RIPA lysis buffer according to the manufacturer's protocol, and the protein concentration was measured using the BCA protein assay kit. The GAG was extracted from the total protein by precipitating in ethanol (95%)/sodium acetate (1.3%) aqueous solution and resolved in saline. Then, 50 µL of GAG solution was mixed with 200 µL of DMMB solution that contained 16 µg/L DMMB, 3.04 mg/mL glycine, 2.37 mg/L NaCl (pH = 3.0), and obtained an OD at the wavelength of 525 nm. The GAG content was determined according to a standard curve of chondroitin sulfate.

### 5.23 Evaluation of In Vivo Therapeutic Effect of Magneto‐Mechanical Modulation on Cartilage Defect

Animal protocols were approved by the Institutional Animal Care and Use Committee of Tongji University (Approval number: TJAA07325201), and all animal procedures were performed in accordance with the ARRIVE (Animal Research: Reporting of In Vivo Experiments) guidelines 2.0. SD male rats (RRID: MGI:5651135, 8 weeks, ∼300 g) were selected to create a cartilage defect model in both hind legs. Specifically, the rat was anaesthetized by intraperitoneal injection of pentobarbital sodium (1%, 40 mg/ kg), and the knee joint was opened to expose the distal femur. Bilateral femoral defects with a size of 2 mm × 2 mm (diameter × height) were then created on the trochlear grooves by a drill. For microfracture, several punctures were created on the defect region using a needle to release the bone marrow. This animal model was used in the following experiments. During the experiments, the body weight of the rats was collected (Table S2 and s3) to ensure that no animal experienced weight loss. For the in vivo BMSC recruitment experiment, sixteen rats were randomly divided into four groups (n = 4 for 7 or 14 days), namely TGel group, PDA@RFNM DS group, E7@RFNM DS group with no MF, and 3D MF actuation. TGels or NMDS were injected into the defects with a syringe post microfracture. After the wound was sutured, the rats were allowed to move freely. In the following 14 days, the rats in the MF actuation group were exposed to the 3D MF (75 mT, [2, 1] Hz, 10 min/day) every day. On the eighth and 15th day, the rats were euthanized, and the femurs were collected and fixed with paraformaldehyde solution (4%). Immunofluorescence staining for MSC‐specific markers CD90 (Proteintech Cat# 27178‐1‐AP, RRID: AB_3085933) and CD105 (Proteintech Cat# 10862‐1‐AP, RRID: AB_2098906) was performed with serial sections of the femoral samples to determine the effect of magneto‐actuated NMDS on BMSCs mobilization in vivo. For the long‐term cartilage repair experiments, twenty‐one rats were randomly divided into seven groups (n = 6), namely, Sham group, Defect group, Microfracture groups with no MF and 3D MF actuation, Microfracture + E7@RFNM DS groups with no MF, 2D MF, and 3D MF actuation. According to the groups, TGels or E7@RFNM DS were injected into these defects with a syringe after the model was created. After the wound was sutured, the rats were allowed to move freely. In the following 30 days, the rats in MF actuation groups were exposed to the 2D MF (75 mT, 2 Hz, 10 min/day) or 3D MF (75 mT, [2, 1] Hz, 10 min/day) every day. On the 31st day, the rats were euthanized, and the femurs were collected for gross observation. After fixed, serial sections of the femoral samples were stained with H & E, SO & FG, and PB & NR for histological analysis. The ICRS cartilage repair assessment system (Table S4) was also used to evaluate the curative effects of cartilage repair.

### 5.24 Statistical Analysis

All quantitative results are presented as the mean ± standard deviation. A two‐tailed Student's *t*‐test was used for statistical analysis when comparing two groups. One‐way analysis of variance (ANOVA) with Tukey's multiple comparisons test was performed to make pairwise comparisons between multiple groups. A p‐value less than 0.05 was indicated statistically significant.

## Author Contributions

Conceptualization: Z.L. and L.P.; Methodology: Z.L., L.P., and L.L.; Investigation: Y.W.; Visualization: X.C.; Supervision: C.D. and Y.C.; Writing – original draft: Z.L.; Writing – review & editing: F.Y., C.D., and Y.C.

## Funding

National Key Research and Development Program of China (No. 2021YFA1201400), National Natural Science Foundation of China (No. 32271441), Shanghai Municipal Education Commission Innovative Program (No. 2023ZKZD27), Shanghai Pilot Program for Basic Research (No. 15002360232), Tongji University Medicine‐X Interdisciplinary Research Initiative (No. 2025‐0554‐YB‐18), China Postdoctoral Science Foundation (No. 2024M762433).

## Conflicts of Interest

The authors declare no conflicts of interest.

## Supporting information




**Supporting File 1**: advs75958‐sup‐0001‐SuppMat.docx.

**Supporting File 2**: advs75958‐sup‐0002‐MovieS1.mp4.

**Supporting File 3**: advs75958‐sup‐0003‐MovieS2.mp4.

**Supporting File 4**: advs75958‐sup‐0004‐MovieS3.mp4.

**Supporting File 5**: advs75958‐sup‐0005‐MovieS4.mp4.

## Data Availability

The data that support the findings of this study are available from the corresponding author upon reasonable request.
