# Supplementary material for: A Wireless 3D Magneto‐Mechanical Stimulation Platform Drives In Situ Chondrogenic Commitment of Endogenous MSCs
Source: Adv Sci (Weinh). 2026 Jun 4;13(48):e75958. doi: 10.1002/advs.75958 (PMC13337046; doi:10.1002/advs.75958)
Supplement: Supplementary file 1 [file ADVS-13-e75958-s002.docx]

Supporting Information

A wireless 3D magneto-mechanical stimulation platform drives in situ chondrogenic commitment of endogenous MSCs

Zhenguang Li, Li Peng, Laiya Lu, Yingying Wang, Xiaolei Chen, Feng Yin, Cuijun Deng*, Yu Cheng*

* Corresponding author.

Email: cuijundeng@tongji.edu.cn (C. Deng), yucheng@tongji.edu.cn (Y. Cheng)

**This file includes:**

Supplementary Text

Figs. S1 to S18

Tables S1 and S4

**Other Supplementary Materials for this manuscript include the following:**

Movies S1 to S4

**Supplementary Text**

***Materials***

Rhein (≥ 98% AR), ethylene glycol (EG, > 99% GC), polyethyleneimine (PEI, ≥ 99%, M.W. = 600), polyvinyl pyrrolidone (PVP, GR, K30), ferric chloride hexahydrate (FeCl_3_·6H_2_O, 99% AR), sodium acetate anhydrous (NaAc, 99.9% GR), dopamine hydrochloride (≥ 98% AR), 1,1-diphenyl-2-picrylhydrazyl (DPPH, ≥ 98.5% HPLC) and 1,9-dirnethyl-methylene blue (DMMB, 80%) were purchased from Shanghai Aladdin Biochemical Technology Co., Ltd. E7 peptide (EPLQLKM) and FITC-labeled E7 peptide (FITC-E7) were obtained from GL Biochem (Shanghai) Ltd. Sodium hyaluronate (HA, 20 ~ 40 kDa) was supplied by Tianjin Heowns Biochemical Technology Co., Ltd. Pluronic® F-127 (BioReagent), crystal violet, and bovine serum albumin (BSA) were provided by Sigma-Aldrich. Penicillin-streptomycin (Gibco, 15140122) and trypsin-EDTA (0.25%, Gibco) were purchased from Thermo Fisher Scientific Inc. Cell counting kit-8 (CCK-8, Biosharp) was obtained from Labgic Technology Co., LTD.

***Finite element simulation***

Finite element method (FEM) was conducted using COMSOL Multiphysics 6.3. The model primarily utilized the Computational Fluid Dynamics (CFD) module, specifically integrating the Laminar Flow interface with the Dynamic Mesh feature to investigate the variations in flow velocity induced by the rotation of the nanomotors All computations were performed on a workstation equipped with an AMD Ryzen 5 5600G CPU (@3.9 GHz) and 16 GB of RAM.

***Preparation and characterizations of nanomotor delivery system (NMDS)***

The TGel precursor was prepared by the simply mixing HA (1%) and F-127 (20%) in PBS (pH = 7.4) at 4 ℃ to obtain a homogeneous solution. Then, different concentrations of E7@RFNMs were evenly dispersed in the precursor solution and stored at 4 ℃. The E7@RFNM-loaded hydrogel precursor was warmed to 37 ℃ to realize the sol-gel transition, forming the injectable NMDS for further experiments (Figure S11A). The morphology of NMDS was observed under a transmission electron microscope (TEM, Jeol JEM-1230, Japan) at 5 kV after freeze-drying. The rheological properties of NMDS were measured by a rheometer (Thermo HAAKE MARS 60, USA). The storage modulus (G’) and loss modulus (G’’) of the hydrogel were recorded at time sweep mode, temperature sweep mode and frequency sweep mode, respectively. The time sweep test (frequency: 1 Hz; strain: 1 %) was performed to observe the sol-gel transition when the temperature rose from 4 ℃ to 37 ℃. The temperature sweep test (frequency: 1 Hz; strain: 1 %) was performed from 10 ℃ to 37 ℃ to determine the gelation temperature. The frequency sweep test was performed from 0.1 Hz to 100 Hz (strain: 1 %) and the viscosity of the hydrogel was recorded synchronously in this mode.

***Characterization analysis of the NMDS***

The hydrogels showed a typical reversible sol-gel transition in cycles between 4 ℃ and 37 ℃ both before and after loading the E7@RFNMs (Figure S11, B and C). Scanning electron microscopy (SEM) observation showed that the TGel possessed a highly interconnected porous structure, and the porous structure was maintained after loading the E7@PFNMs (Figure S11, D and E). The local enlarged SEM image displayed noticeable nanoparticle deposition on the pore-walls of NMDS compared to the smooth pore-walls of TGel, confirming the successful encapsulation of E7@PFNMs in the NMDS (Figure S11, F and G). Additionally, the NMDS could be smoothly extruded through a 26-gauge syringe needle at 37 ℃ without clogging (Figure S12, A and B). The rheological behavior at the time sweep mode showed that the NMDS underwent a rapid sol-gel transition as the storage modulus (G’) gradually exceeded the loss modulus (G’’) when the temperature rose from 4 ℃ to 37 ℃ (Figure S12C). At the temperature sweep mode, both the G’ and G’’ of the NMDS dramatically increased, and G’ became higher than G’’ after ~27 °C, reflecting the critical temperature of gelation process (Figure S12D). The NMDS exhibited an elastic feature with the G’ remaining higher than the G’’ at the frequency sweep mode (Figure S12E). And the viscosity of the NMDS decreased continuously with the increase of shear frequency, revealing the shear-thinning behavior of injectability (Figure S12F).

***Cytocompatibility evaluation of the NMDS***

After BMSCs were incubated for 24 h, different concentrations of E7@RFNMs loaded NMDS were injected to cover the cells. After incubation for 1, 3, 5 and 7 days, the cells were incubated with CCK-8/DMEM solution (10%, v/v) for 2 h, and the supernatant was withdrawn to measure the OD at the wavelength of 450 nm. Inoculated wells without hydrogels were used as control. Besides, the cells at day 1 were stained by Calcein/PI Live/Dead assay kit (Beyotime, C2015, China) according to the manufacturer’s instructions and imaged on a cell imaging system.

***Hemolytic activity evaluation of the NMDS***

The fresh anticoagulant rat blood was centrifuged at 2000 rpm for 10 min, washed thrice with PBS, and diluted with PBS to obtain 10% (v/v) of red blood cell (RBC) suspension. Then, different concentrations of E7@RFNMs loaded NMDS were mixed with RBC suspension (200 μL), and made up to 1 mL with PBS. After incubation at 37 ℃ for 4 h, the mixed solutions were centrifuged (2000 rpm, 10 min) and photographed. Finally, 100 μL of each supernatant was withdrawn to measure the OD at the wavelength of 540 nm. The untreated RBC suspensions diluted with PBS and deionized water, respectively, were taken as the negative control (NC) and the positive control (PC). The hemolytic rates were calculated by the following equation:

Hemolytic rate (%) = (OD_Sample_ – OD_NC_)/(OD_PC_ – OD_NC_) × 100	(S1)

***Analysis of the cytocompatibility and hemocompatibility of the NMDS***

The compatibility of the NMDS loading different concentrations of E7@RFNMs with BMSCs was investigated. After 24 h of coculture, all groups did not cause obvious cell death with the absence of visible propidium iodide (PI) signal, although the NMDS loading ≥ 40 μg/mL of E7@RFNMs showed a slight disadvantage in BMSCs survival (> 80% of relative survivals) from the quantitative calcein fluorescence results (Figure S13, A and B). Extending the coculture up to 7 days, no significant difference was found in cell proliferation among the groups when the concentration of E7@RFNMs was < 160 μg/mL (Figure S13C). Besides, the hemolysis assay showed that the NMDS possessed excellent hemocompatibility with very low of hemolytic rates (< 5%), indicating high biosafety for *in vivo* applications (Figure S14).

***Intracellular ROS scavenging and cytoprotection evaluation of the NMDS***

For intracellular ROS scavenging evaluation, BMSCs were cocultured with different concentrations of E7@RFNMs loaded NMDS for 24 h, and then the cells were treated with H_2_O_2_ (200 µM) for 30 min. Next, the ROS-specific probe DCFH-DA (Beyotime, S0033, China) and a nucleus dye Hoechst (33258, Beyotime, China) were added according to the manufacturer’s manual to incubate for another 30 min. The fluorescent images were captured with argon laser lines of 405 nm and 488 nm on a confocal laser scanning microscope. The fluorescence intensity of images was analyzed by the ImageJ software.

For the cytoprotection evaluation, BMSCs were cocultured with different concentrations of E7@RFNMs loaded NMDS for 24 h, and then treated with H_2_O_2_ (200 µM) for another 24 h. After the cells were incubated with CCK-8/DMEM solution (10%, v/v) for 2 h, the supernatant was withdrawn to measure the OD at the wavelength of 450 nm on a microplate reader. The relative cell viability was calculated taken the OD of the control group as 100%.

***Evaluation of inflammation-related gene expression of BMSCs on the NMDS***

BMSCs were cocultured with NMDS for 24 h, and then exposed to the 3D MF (75 mT, [2,1] Hz) or not for 3 days (10 min/day). During this period, the complete medium containing IL-1β (10 ng/mL) was used and refreshed every day. Finally, the cells were collected for detecting the expression of inflammation-related genes (*Il-1β*, *Il-6*, *Il-4* and *Il-10*) by RT-qPCR. The sequences of primer pairs were shown in Table S1.

***Analysis of the cytoprotection and inflammation regulation of the NMDS***

The NMDS inherited the antioxidative properties of nanomotors (Figure S17, A and B), and was believed to reduce ROS level through the release of nanomotors, thereby protecting BMSCs from oxidative damage and suppressing the inflammatory microenvironment (Figure S17C). The protective effects of the NMDS on BMSCs were investigated using 200 μM of H_2_O_2_ to mimic biological ROS environment. The results showed a gradual elevating trend in cell viability from ~80% to ~95% with increasing PDA@RFNM or E7@RFNM concentrations from 40 μg/mL to 160 μg/mL in the NMDS, and they were remarkably higher than the cell viability (< 70%) in the nanomotor-free groups (Figure S17D). The intracellular ROS level of BMSCs cultured in the NMDS was visualized with the ROS-specific probe DCFH-DA. The DCF fluorescence intensity in BMSCs decreased as the concentrations of PDA@RFNMs or E7@RFNMs in the NMDS increased, and was significantly lower than that in the positive group (Figure S17, E and F). And the NMDS containing PDA@RFNMs and E7@RFNMs did not show obvious differences in DCF fluorescence intensity due to the restoring antioxidative ability after E7 peptide released (Figure S17G). The inflammation-related gene expression of BMSCs cultured in the NMDS was further studied by using 10 ng/mL of IL-1β to create an inflammation-mimicking environment. In contrast to the BMSCs in the TGel groups, the BMSCs in the NMDS groups significantly downregulated the expression of pro-inflammatory genes (*Il-1β* and *Il-6*) and upregulated the expression of anti-inflammatory genes (*Il-4* and *Il-10*), regardless of the 3D MF actuation (Figure S17, H-K).

Figure S1. Preparation and characterizations of nanomotors. (A) Schematic illustration of the synthesis and modification process for nanomotors. (B) Representative TEM images of RFNMs. (C) Statistical size of E7@RFNMs from TEM images. (D, E) Hydrodynamic diameters and zeta potentials of nanomotors. (F) Wide scan XPS spectra of nanomotors. (G, H) Fe 2p and O 1s high-resolution spectra of E7@RFNMs.

Figure S2. Antioxidative activity of nanomotors. (A) Radical scavenging mechanism of nanomotors for antioxidative activity. (B) Photographs of DPPH· after reaction with different concentrations of nanomotors. (C-E) UV-vis spectra of DPPH· after reaction with different concentrations of RFNMs, PDA@RFNMs and E7@RFNMs.

Figure S3. (A) Photograph of the appearance of the magnetic actuation system. (B) Assembly behavior of nanomotors under the actuation of the 3D MF (20 mT, [1,1] Hz). (C) Statistical assembly length of nanomotors over time.

Figure S4. (A) Prussian blue-nucleus fast red staining of BMSCs after cocultured with different concentrations of nanomotors for 24 h. (B) Quantitative uptake of nanomotors by BMSCs at different concentrations (n = 3, ****P* < 0.001). (C) Labeling efficiency of nanomotors on BMSCs.

Figure S5. (A) Relative cell viability of BMSCs after cocultured with different concentrations of nanomotors for 24 h. (B) Proliferation of BMSCs after cocultured with different concentrations of E7@RFNMs for 1, 3, 5 and 7 days (n = 6, ****P* < 0.001). (C) Crystal violet staining of BMSCs after cocultured with different concentrations of E7@RFNMs for 1, 3, 5 and 7 days.

Figure S6. Proliferation of BMSCs labeled with 5 μg/mL (A), 10 μg/mL (B) and 20 μg/mL (C) of E7@RFNMs under the actuation of different frequencies of 3D MF (75 mT, 10 min/day) for 1, 3, 5 and 7 days (n = 6, ***P* < 0.01 and ****P* < 0.001).

Figure S7. (A) The expression of chondrogenic genes of BMSCs labeled with different concentrations of E7@RFNMs under the actuation of different frequencies of 3D MF (75 mT, 10 min/day) for 3 days (n = 3, ****P* < 0.001). (B) The expression of chondrogenic genes of BMSCs labeled with 10 μg/mL of E7@RFNMs under the 3D MF actuation (75 mT, [2,1] Hz) with different duration time per day for 3 days (n = 4, **P* < 0.05 and ****P* < 0.001).

Figure S8. The expression of chondrogenic genes of BMSCs labeled with 10 μg/mL of E7@RFNMs in complete and chondrogenic medium under the 3D MF actuation (75 mT, [2,1] Hz, 10 min/day) for 3 days (n = 4, **P* < 0.05, ***P* < 0.01 and ****P* < 0.001).

Figure S9. (A) COL Ⅱ immunofluorescence staining of BMSCs labeled with 10 μg/mL of E7@RFNMs in complete or chondrogenic medium under the 3D MF actuation (75 mT, [2,1] Hz, 10 min/day) for 7 days. (B) Quantitative average fluorescence intensity by the immunofluorescence staining images (n = 6, **P* < 0.05 and ***P* < 0.01).

Figure S10. (A) Fluorescence images of BMSCs labeled with 10 μg/mL of E7@RFNMs and stained with Lyso-tracker Red/Hoechst. The green tracks of lysosome movement were present by ImageJ software. (B) Quantitative lysosome displacement within 5 minutes under the 3D MF actuation (n > 35, **P* < 0.05 and ****P* < 0.001).

Figure S11. (A) Schematic illustration of the formation process of the E7@RFNM-loaded thermosensitive hydrogel delivery system. (B) Optical images of the TGel formed by mixing HA and F-127 undergoing the sol-gel transition in the cycles between 4 ℃ and 37 ℃. (C) Optical images of the NMDS formed by mixing E7@RFNMs and TGel undergoing the sol-gel transition in the cycles between 4 ℃ and 37 ℃. (D-G) SEM images of the lyophilized TGel and NMDS at different magnifications.

Figure S12. (A, B) Optical images of the NMDS extruded onto a galss and injected into water by a 26-gauge needle at 37 ℃. (C-E) Rheological behaviors of the NMDS at dynamic time sweep mode, temperature sweep mode and frequency sweep mode. (F) Changes in viscosity of the NMDS with the shear frequency.

Figure S13. (A) Calcein-AM/PI staining of BMSCs after cocultured with different concentrations of E7@RFNMs loaded NMDS for 24 h. (B) Quantitative cell survival by the Calcein-AM/PI staining (n = 6, ***P* < 0.01). (C) Proliferation of BMSCs after cocultured with different concentrations of E7@RFNMs loaded NMDS for 1, 3, 5 and 7 days (n = 6, **P* < 0.05 and ***P* < 0.01).

Figure S14. Optical images and corresponding hemolytic rates of E7@RFNMs (A), TGels (B), and NMDS (C) at different concentrations after contact with the RBC suspension.

Figure S15. (A) TEM images of the nanomotors released at day 7. (B) Crystal violet staining of BMSCs cocultured with the NMDS migrating towards the scratch under the 3D MF actuation (75 mT, [2,1] Hz, 10 min/day). (C) Quantitative wound healing percentage by the crystal violet staining (n = 5, **P* < 0.05 and ****P* < 0.001).

Figure S16. PB & NR staining of femoral sample sections after the NMDS implantation for 7 and 14 days.

Figure S17. (A) UV-vis spectra of DPPH· after reaction with different concentrations of E7@RFNMs loaded NMDS. (B) Quantitative DPPH· scavenging rates of different NMDS. (C) Schematic illustration of nanomotors released from the NMDS reducing ROS levels and protecting BMSCs. (D) Relative cell viability of BMSCs cocultured with the NMDS after treating with 200 μM H_2_O_2_ for 24 h (n = 6, ****P* < 0.001). (E, F) DCF fluorescence images of BMSCs cocultured with the NMDS containing different concentrations of E7@RFNMs and PDA@RFNMs after treatment with 200 μM H_2_O_2_. (G) Quantitative fluorescence intensity from DCF fluorescence images of BMSCs (n = 3). (H-K) The expression of inflammation-related genes of BMSCs cocultured with the NMDS under the 3D MF actuation. 10 ng/mL of IL-1β was used to create an inflammation-mimicking environment (n = 4, **P* < 0.05 and ****P* < 0.001).

Figure S18. Representative macroscopic photograph of the joint sample and H & E staining, SO & FG staining and PB & NR staining of femoral sample sections at Sham group.

Table S1. The primer sequences used for RT-qPCR analysis

| Gene | Forward primer | Reverse primer |
| --- | --- | --- |
| *Gapdh* | 5’- ACTCCCATTCTTCCACCT | 5’- CCTGTTGCTGTAGCCATA |
| *Col2a1* | 5’- CTCAAGTCGCTGAACAACCA | 5’- GTCTCCGCTCTTCCACTCTG |
| *Sox9* | 5’- CTGAAGGGCTACGACTGGAC | 5’- TACTGGTCTGCCAGCTTCCT |
| *Acan* | 5’- CAGGGATAACGGACTGAA | 5’- GAGTAAAGTGGTCATAGTTCAG |
| *Il-1β* | 5- ATCTCACAGCAGCATCTCGA | 5’-TAGCAGGTCGTCATCATCCC |
| *Il-6* | 5’- ACTTCCAGCCAGTTGCCTTCTTG | 5’-TGGTCTGTTGTGGGTGGTATCCTC |
| *Il-4* | 5’- CAAGGAACACCACGGAGAACGAG | 5’- TTCTTCAAGCACGGAGGTACATCAC |
| *Il-10* | 5’- GCATCCAGACACACACAGACTAGAC | 5’- GCCCAGAGACAGACAAGCAAGAG |

Table S2. The body weight of rats collected at initial and end point during the BMSC recruitment experiments

|  | Weight at day 0 (g) | Weight at day 8 (g) |
| --- | --- | --- |
| TGel | 310.8 | 315.2 |
|  | 331.7 | 331.6 |
| PDA@RFNM DS | 315.9 | 318.6 |
|  | 328.9 | 329.5 |
| E7@RFNM DS | 321.8 | 330.2 |
|  | 314.7 | 318.5 |
| E7@RFNM DS + 3D MF | 316.7 | 326.7 |
|  | 325.4 | 333.2 |
|  | Weight at day 0 (g) | Weight at day 15 (g) |
| TGel | 309.7 | 320.8 |
|  | 318.9 | 334.1 |
| PDA@RFNM DS | 314.4 | 331.4 |
|  | 320.6 | 342.7 |
| E7@RFNM DS | 317.8 | 332.5 |
|  | 322.1 | 335.6 |
| E7@RFNM DS + 3D MF | 307.4 | 316.6 |
|  | 314.5 | 327.9 |

Table S3. The body weight of rats collected at initial, intermediate and end point during the cartilage repair experiments

|  | Weight at day 0 (g) | Weight at day 15 (g) | Weight at day 31 (g) |
| --- | --- | --- | --- |
| Sham | 296.7 | 324.4 | 337.9 |
|  | 289.3 | 312.7 | 329.7 |
|  | 306.8 | 325.8 | 339.6 |
| Defect | 304.6 | 327.4 | 338.9 |
|  | 306.4 | 325.8 | 335.4 |
|  | 295.5 | 318.9 | 335.7 |
| Microfracture | 299.8 | 321.6 | 338.6 |
|  | 313.5 | 330.1 | 341.6 |
|  | 296.2 | 320.5 | 334.2 |
| Microfracture + 3D MF | 295.2 | 326.8 | 339.4 |
|  | 315.2 | 332.6 | 345.2 |
|  | 291.3 | 314.7 | 332.6 |
| Microfracture + E7@RFNM DS | 288.6 | 310.5 | 328.5 |
|  | 296.7 | 323.9 | 337.9 |
|  | 306.5 | 327.7 | 337.2 |
| Microfracture + E7@RFNM DS + 2D MF | 297.8 | 328.7 | 340.5 |
|  | 294.6 | 324.6 | 338.3 |
|  | 305.2 | 326.8 | 337.8 |
| Microfracture + E7@RFNM DS + 3D MF | 310.5 | 330.8 | 346.7 |
|  | 306.4 | 325.6 | 338.9 |
|  | 289.6 | 316.4 | 333.1 |

Table S4. International Cartilage Repair Society (ICRS) cartilage repair assessment

| Criteria | | Points |
| --- | --- | --- |
| Degree of defect repair | In level with surrounding cartilage | 4 |
|  | 75% repair of defect depth | 3 |
|  | 50% repair of defect depth | 2 |
|  | 25% repair of defect depth | 1 |
|  | 0% repair of defect depth | 0 |
| Integration to border zone | Complete integration with surrounding cartilage | 4 |
|  | Demarcating border < 1mm | 3 |
|  | 3/4 of graft integrated, 1/4 with a notable border >1mm width | 2 |
|  | 1/2 of graft integrated with surrounding cartilage,  1/2 with a notable border > 1mm | 1 |
|  | From no contact to 1/4 of graft integrated with surrounding cartilage | 0 |
| Macroscopic appearance | Intact smooth surface | 4 |
|  | Fibrillated surface | 3 |
|  | Small, scattered fissures or cracks | 2 |
|  | Several, small or few but large fissures | 1 |
|  | Total degeneration of grafted area | 0 |
| Overall repair assessment | Grade Ⅰ normal | 12 |
|  | Grade Ⅱ nearly normal | 11-8 |
|  | Grade Ⅲ abnormal | 7-4 |
|  | Grade Ⅳ severely abnormal | 3-1 |

Movie S1.

Motion control of nanomotors under the actuation of the 2D MF (20 mT, 1 Hz).

Movie S2.

Motion control of nanomotors under the actuation of the 3D MF (20 mT, [1,1] Hz).

Movie S3.

Visualized motion of nanomotors inside lysosomes of BMSCs under the actuation of the 2D MF (20 mT, 1 Hz).

Movie S4.

Visualized motion of nanomotors inside lysosomes of BMSCs under the actuation of the 3D MF (20 mT, [1,1] Hz).
